# Surface Fluorination of Nuclear Graphite Exposed to Molten 2LiF–BeF_2_ (FLiBe) Salt and Its Cover Gas at 700 °C

**DOI:** 10.1021/acsaenm.3c00764

**Published:** 2024-06-17

**Authors:** L. Vergari, H. Wu, R. O. Scarlat

**Affiliations:** † Department of Nuclear Engineering, University of California Berkeley, 2521 Hearst. Ave, Berkeley, California 94720, United States; ‡ Department of Nuclear, Plasma and Radiological Engineering, University of Illinois Urbana—Champaign, 104 S. Wright Street, Urbana, Illinois 61801, United States; § Department of Engineering Physics, University of Wisconsin—Madison, 1500 Engineering Drive, Madison, Wisconsin 53706, United States; ∥ Canadian Nuclear Laboratories, 286 Plant Road, Chalk River, Ontario K0J 1J0, Canada

**Keywords:** FLiBe, nuclear graphite, high-temperature fluorination, molten salt reactors, XPS analysis

## Abstract

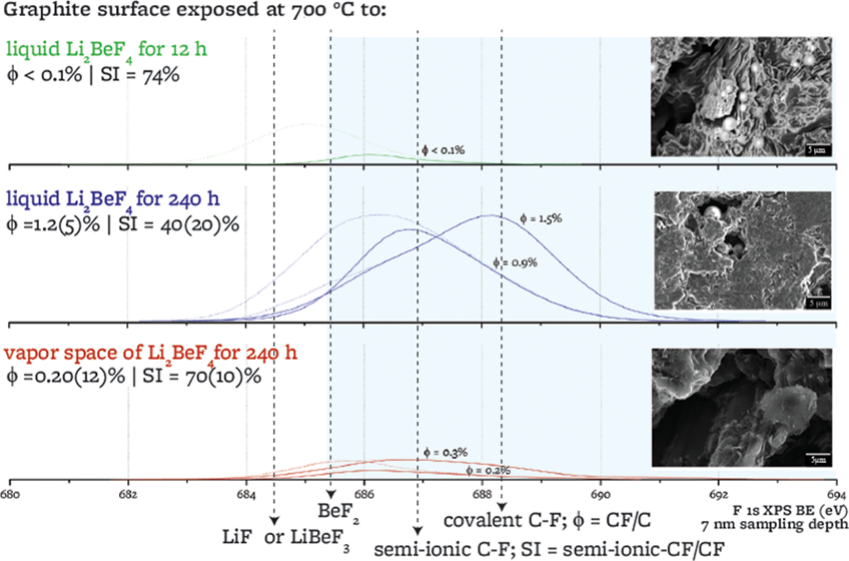

This study demonstrates that the reaction of Li_2_BeF_4_ (FLiBe) with graphite both in the liquid phase and the gas phase of the molten salt leads to the formation of covalent and semi-ionic carbon–fluorine bonds at the graphite surface and is accompanied by surface microstructural changes, removal of C–O groups, and deposition of metallic beryllium, based on XPS, Raman, and glow discharge mass spectroscopy characterization. At 700 °C, the observed surface density of C–F is higher after 240 h than after 12 h of exposure to molten FLiBe salt; the kinetics of covalent C–F formation is slower than that of semi-ionic C–F formation, and the relative amount of semi-ionic C–F content increases with depth. The graphite sample exposed to the cover gas exhibits less surface fluorination than the salt-exposed sample, with predominantly semi-ionic C–F. Based on these observations and the observed LiF/BeF_2_ ratio by surface XPS, the hypotheses that fluorination of the salt-exposed graphite occurs via a gas-phase mechanism or that it requires salt intrusion are refuted; future studies are warranted on the transport of C–F semi-ionic and covalent species in graphite at high temperatures.

## 1 Introduction

Nuclear reactors employ graphite exposed to molten fluoride salts at temperatures of 500 to 800 °C for durations up to several tens of years. Characterizing the chemical interactions at the salt-graphite interface is of relevance to assess the performance of graphite during reactor operation and to predict graphite conditions upon discharge from the reactor. In particular, it is of relevance to understand the impact of graphite-salt chemical interactions on graphite capacity to uptake tritium,^1−4^ on resistance to molten salt infiltration and oxidation,^5,6^ and on the evolution of tribological properties.^7,8^ Advancing the understanding of the chemical interactions between molten Li_2_BeF_4_ (FLiBe) salt and nuclear graphite can help develop predictive models of graphite behavior over several decades of exposure to high-temperature molten salt and neutron irradiation at varying temperatures.

In advanced nuclear reactors, graphite components are present in the cores of fluoride-salt-cooled high-temperature reactors (FHRs) and molten salt reactors (MSRs) with large surface areas exposed to molten salts.^9,10^ For example, the core of the Mark-I FHR contains 2065 m^2^ of graphite surface (688,000 graphite pebbles, outer and inner reflectors) in 12 m^3^ of FLiBe salt,^11^ corresponding to a graphite surface area to salt volume ratio of 172 m^–1^; the molten salt breeder reactor (MSBR) design had 330 m^2^ of graphite moderator surface area in 2.3 m^3^ of fuel salt,^12^ corresponding to a graphite surface area to salt volume ratio of 143 m^–1^. During FHR operations, graphite pebbles and reflectors are exposed to both molten 2LiF–BeF_2_ (FLiBe) and to the cover gas above the salt (e.g., in the defueling chute) cyclically for durations of tens of days to tens of years.

During the molten salt reactor experiment (MSRE), exposure to fuel salt (nominally composed of LiF, BeF_2_, ZrF_4_, UF_4_, and ThF_4_ and also containing UF_3_, fission products, and transuranic elements^13^) for 2.5 years was concluded to lead to “no attack by salt”, citing no change in surface finish and no development of cracks.^13^ This is not a surprising engineering observation, given that for infinite, defect-free graphite, chemical oxidation by FLiBe or by MSRE fuel salt at a UF_4_/UF_3_ redox potential ratio of 10–100 (corresponding to 10^–43^ to 10^–45^ Pa F_2_ partial pressure, i.e., 710 to 740 kJ/mol fluorine potential^14^) is not thermodynamically favorable. For example, C + 2F_2_ = CF_4_ has Δ*G*_f_ = −400 kJ/mol F_2_ at 600 °C;^15^ so, at the MSRE fluorine potential, a partial pressure of 10^–18^ to 10^–21^ Pa of CF_4_ would be expected, indicating a negligible reaction progression from an engineering perspective.

Nevertheless, carbon structures fluorinate at high fluorine potentials (e.g., under 1 atm F_2_ or HF gas),^16−27^ and highly fluorinated graphite is broadly synthesized and studied.^28−34^ Similarly, fluorination of graphite oxide with fluorine gas is known to occur as well.^35−37^ Fluorinated graphite and graphite oxide are examples of high heterogeneous atom content (e.g., units to tens of F or O atomic percent) in the graphite. For MSR and FHR applications, very low fluorine potentials producing low heterogeneous atom content (>100 ppm) are expected. There is previous evidence of fluorination of graphite upon exposure to both FLiBe and LiF–NaF–KF (FLiNaK) molten fluoride salt. In ref (38), formation of C–F bonds replacing pre-existing C–H bonds in nuclear graphite (IG-110, Toyo Tanso Co. Ltd.) exposed to FLiNaK at 500 °C for 16 h by X-ray near edge absorption spectroscopy (XANES) was observed. Reference (15) showed evidence of fluorination of IG-110 after exposure to molten FLiBe at 700 °C for 12 h, based on glow discharge mass spectroscopy (GDMS) that indicated higher penetration in the sample for fluorine compared to beryllium and lithium, and X-ray photoelectron spectroscopy (XPS) that showed a signal for fluorine-bound carbon. Reference (39) used laser-induced breakdown spectroscopy (LIBS) to study FLiBe and FLiNaK penetration in graphite, observing evidence of a possible reaction of KF and graphite upon exposure to 10 bar FLiNaK at 750 °C for 12 h.

A mechanistic description of the surface fluorination of graphite by FLiBe has not yet been developed. For example, graphite has porosity on the scale of several to tens of microns,^40^ and FLiBe is nonwetting on graphite.^5^ It is not known if fluorination of the salt-exposed graphite proceeds via a gas-phase reaction, and thus fluorination should be expected at the entire surface of the open porosity, or if it proceeds predominantly at liquid salt-graphite interface and thus should be expected to be present strictly at the surface of the sample. It is also not yet known if fluorination will proceed to higher levels of surface C–F concentration upon longer exposures or if the graphite surface has already reached chemical equilibrium with the salt within 12 h of exposure considered in ref (15). Understanding the nature of the reaction kinetics is of engineering relevance if the performance of the graphite surface under molten salt is to be predicted for one to tens of years of in-salt operation.

To answer these fundamental questions about the mechanism and the kinetics of the surface fluorination of graphite, we perform a longer (240 h) exposure at 700 °C, with samples exposed to the liquid salt, and to the cover gas above it, with an approximate graphite surface area to salt volume of 100 m^–1^. Samples are characterized by surface and depth profiling using XPS, Scanning Electron Microscopy and Energy Dispersive X-ray Spectroscopy (SEM/EDS), and Raman spectroscopy.

## 2 Experimental Section

### 2.1 Materials

Three samples made of the same source block of IG-110 grade nuclear graphite are used in this study: one sample is kept as a reference, one sample is exposed to liquid FLiBe, and one sample is exposed to the cover gas above the salt. The graphite source block was provided by Dr. Will Windes from Idaho National Laboratory (INL), and Table 1 summarizes the properties of IG-110 grade nuclear graphite. Hydro-fluorinated FLiBe (2.07 ± 0.11:1 molar ratio LiF/BeF_2_) is used in this study, and refs (41−43) describe its preparation and characterization. The same salt batch and graphite source block are used as in ref (15). The impurities in FLiBe and in graphite are reported in Table 2, reproduced from refs^15 and 44^

**Table 1:** Properties of IG-110 Grade Nuclear Graphite, as Reported by the Manufacturer Unless Otherwise Specified

manufacturer	Toyo Tanso Co. Ltd
fabrication process	cold isostatic pressing
bulk density (g/cm^3^)	1.77
open porosity	14–18%^45^
total porosity	22%
average grain size (μm)	20
average pore size (μm)^45^	3

**Table 2:** Impurity Analysis of FLiBe and Graphite Materials Used in This Experiment, in weight ppm, Reproduced from Refs (15 and 44)

FLiBe elemental analysis (wppm), by inductively coupled plasma mass spectrometry (ICP-MS)^15^		IG-110 graphite elemental analysis (wppm), by glow discharge mass spectrometry (GDMS)^44^	
**K**	293(16)	**S**	29(0.1)
**Mg**	103(7)	**Si**	18(0.1)
**Ca**	92(19)	**F**	16(0.1)
**Al**	18.8(1.5)	**Li**	14(0.05)
**Cr**	10.9(1.1)	**K**	7.1(0.1)
**Fe**	6.6(8)	**Al**	6.9(0.1)
**V**	0.38(0.08)	**Ca**	4.5(0.1)
**Ni**	0.3(0.1)	**Cl**	4.1(0.1)
		**V**	3.2(0.01)
		**As**	2.4(0.1)
		**Mg**	1.4(0.1)
		**Cr**	1.1(0.1)
		**Ni**	1.1(0.1)
		**Fe**	0.6(0.1)
		**Be**	0.08(0.05)

### 2.2 Sample Preparation

Table 3 describes the three samples used in this study. The mass of all samples before and after vacuum heat treatment is measured with a QUINTIX224-1S Sartorius analytical balance with built-in internal calibration (220 g range, 0.0001 g readability/repeatability) outside the glovebox. Sample weight change due to vacuum baking is measured for each sample and averages to 0.08(2)%; the errors are calculated by adding instrumental error and measurement error in quadrature.

**Table 3:** Description of Graphite Samples

sample	sample surface	sample preparation	exposure type	dimensions (cm)
ref	Ref_P: polished surface	machined with a low-speed diamond saw, polished with 1200 grit SiC paper one surface, sonicated in DI water for 5 min, and pre-baked in vacuum (in an alumina boat in a tube furnace) at 1500 °C for 12 h, exhibiting a baking mass loss of 0.08(2)%.^a^ Cooled inside the furnace under vacuum and then stored inside an Ar glovebox before the experiment	not exposed	*L × H × W*: 1 × 1 × 0.3
	Ref_M: as-machined surface			
L12^a^	L12_P: polished surface^a^		exposed to molten FLiBe at 700 °C for 12 h in a positive-pressure Ar glovebox (<1 ppm of O_2_ and H_2_O). After experiment, kept for 3 h in cover gas above FLiBe to cool down to room temperature. Sample is discussed in ref (15)	
L240	L240_P: polished surface		exposed to molten FLiBe at 700 °C for 240 h in a positive-pressure Ar glovebox (<1 ppm of O_2_ and H_2_O). After experiment, kept for 3 h in cover gas above FLiBe to cool down to room temperature	
	L240_M: as-machined surface			
G240	G240_P: polished surface		exposed to cover gas above FLiBe at 700 °C for 240 h in a positive-pressure Ar glovebox (<1 ppm of O_2_ and H_2_O). After experiment, kept for 3 h in cover gas above FLiBe to cool down to room temperature	
	G240_M: as-machined surface			

a Sample L12 is pre-baked in a stainless steel vessel in Ar gas at 1000 °C for 3 h, exhibiting a baking mass loss of 0.029(8)%.

**Table 4:**
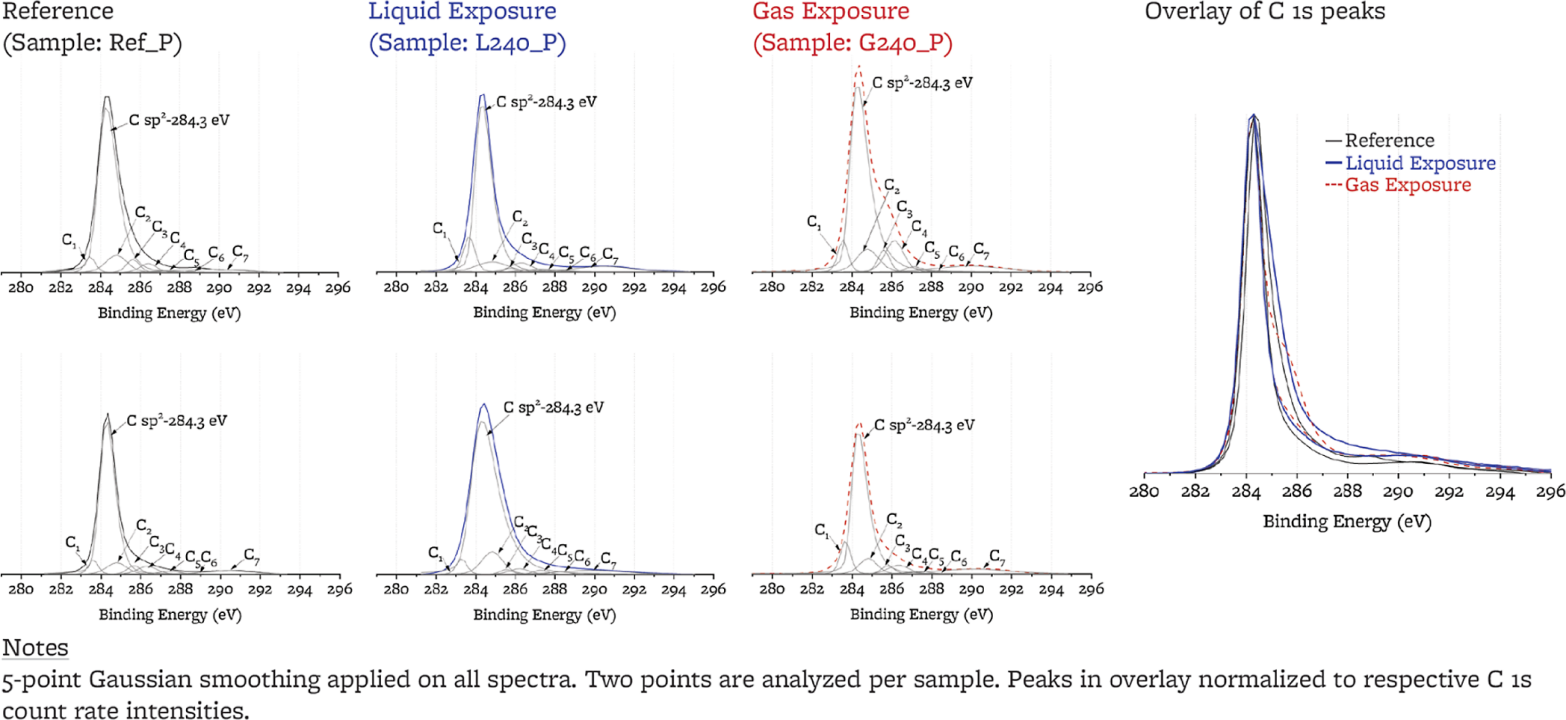
XPS C 1s Peak Fitting for Spectra Collected on the Polished Surfaces of Reference and Exposed Samples^a^

C 1s sub-peak	BE (eV), normalized to 284.3 eV C 1s peak|FWHM (eV)						C 1s sub-peak interpretation	
	area (%), relative to the total C1s peak area							
	reference (Ref_P)		liquid exposure (L240_P)		gas exposure (G240_P)			
	point 1	point 2	point 1	point 2	point 1	point 2		
C sp^2^	284.3|1.1	284.3|0.9	284.3|0.9	284.3|1.5	284.3|1.1	284.3|0.9	sp^2^	(64,65)
	73.7%	72.3%	66.1%	78.1%	63.1%	63.6%		
C_1_	283.5|0.6	283.6|0.6	283.7|0.7	283.3|0.6	283.5|0.6	283.7|0.6	point defects	(64−66)
	3.7%	4.3%	9.5%	3.0%	5.5%	9.3%		
C_2_	284.8|1.3	284.8|1.2	284.8|1.9	284.8|1.3	284.8|1.3	284.8|1.0	sp^3^	(64,65,67)
	8.6%	7.1%	7.6%	8.8%	8.6%	7.5%		
C_7_	289.4|3.9	290.4|2.8	290.5|3.5	289.9|3.7	290.0|3.8	290.1|4.5	π–π*	(65,67,68)
	5.1%	5.5%	7.7%	4.8%	6.8%	10.0%		
C_3_	285.6|0.8	285.7|0.8	285.8|0.6	285.5|0.7	285.6|0.7	285.8|0.7	OC and FC groups	(24−26,33,34,63,65,68−69,70,71,72)
	4.0%	3.6%	1.0%	1.1%	4.4%	2.8%		
C_4_	286.4|1.0	286.3|1.1	286.3|1.3	286.2|1.1	286.1|1.1	286.3|1.2		
	3.5%	4.8%	4.6%	2.1%	9.9%	5.0%		
C_5_	287.2|1.5	287.4|1.2	287.2|1.8	287.3|0.9	287.1|0.9	287.3|1.3		
	1.1%	2.0%	2.0%	1.1%	1.5%	1.6%		
C_6_	288.5|0.5	288.6|0.7	288.0|1.8	288.2|1.0	288.1|0.8	288.1|1.3		
	0.3%	0.3%	1.4%	0.9%	0.3%	0.2%		
*A*_C3_ + *A*_C4_ + *A*_C5_ + *A*_C6_	8.9%	10.7%	9.0%	5.2%	16.1%	9.6%		
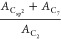	9.2	10.9	9.8	9.4	8.1	9.8	sp^2^/sp^3^ ratio	

a Rows are ordered by type of peak assignment.

### 2.3 Salt Exposure

**Figure 1:**
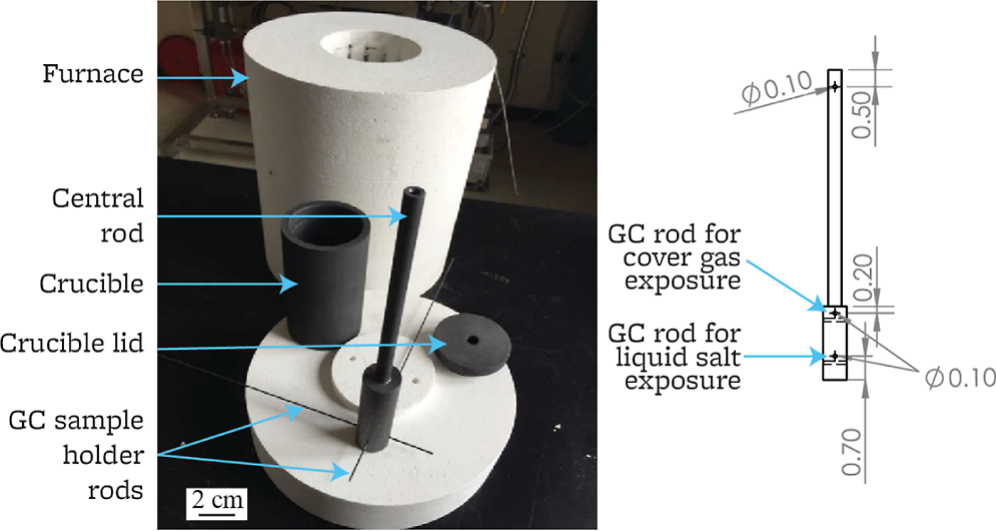
Test apparatus for salt exposure of graphite samples. Graphite area = 0.234 m^2^; salt volume = 0.0022 cm^3^, graphite area to salt volume ratio = 100 m^–1^. Drawing units are cm.

The apparatus used in this experiment is an evolution of the experimental apparatus described in ref (15). The setup consists of a crucible, crucible lid, central rod, and sample-holding rods, to which the samples are connected (Figure 1). To prevent the introduction of metallic impurities or products of metal corrosion in the experimental apparatus, no metallic components are used for the experiment. The crucible and central rod are made from a block of IG-110 graphite provided by Dr. Will Windes at INL. The sample holder rods are made of 2 mm diameter, 100 mm long, type 2 glassy carbon (Alfa Aesar, part 038010-DM). Sample holder rods are located both above and below the salt free surface, to expose graphite samples to both the liquid FLiBe and the cover gas above it.

The experimental apparatus is set inside a vertical furnace, and the experiment is performed inside an inert argon atmosphere glovebox (LC Technology Solutions, Inc.), O_2_ < 1 ppm and H_2_O < 1 ppm, operated at a slight positive pressure (0–10 mbar). The samples are exposed at a temperature of 700 °C for 240 h, as measured by an ungrounded Type K thermocouple (Omega, part KMQXL-040U-12) and read with a data acquisition model (National Instrument, part cRIO-9067) running LabVIEW 2018. At the end of the experiment, the test samples are raised a few centimeters above the salt level at 700 °C; the entire setup is cooled to room temperature over the course of 3 h. Test samples are subsequently removed from the central rod and stored inside the argon glovebox.

### 2.4 Sample Characterization

Full characterization via SEM, EDS, XPS, and Raman is performed for the polished surfaces of the reference and test samples. XPS and Raman spectra are acquired for the as-machined surfaces. Before characterization, samples are sonicated in DI water for 2 min. Characterization with SEM, EDS, and XPS is conducted at University of Wisconsin Madison (UWM). The samples are then packaged according to OSHA standards for beryllium safety (OSHA 29 CFR 1910.1024) and shipped to University of California Berkeley (UCB), where they are stored in an argon atmosphere and characterized via SEM, EDS, and Raman spectroscopy.

#### 2.4.1 Scanning Electron Microscopy and Energy Dispersive X-ray Spectroscopy

Secondary electron SEM micrographs at various magnification levels and EDS maps are collected at four to five different locations, each on the reference sample and on the two exposed samples. Nuclear graphite has a heterogeneous microstructure, with a distribution of sizes of pores, grains, and crystallites. As such, SEM micrographs and EDS maps at each location may not be representative of the full sample and are therefore not used for quantitative calculations.^40,46^ SEM is performed at UCB using a Thermo Fisher Scios 2 with an accelerating voltage of 20 kV and a current of 0.40 nA. Beryllium safety handling procedures are described in Section 2.5. Secondary electrons have an escape depth of approximately 3 nm in graphite.^47^ EDS maps are acquired with Scios 2 at the same voltage and current and analyzed on AZtec 2.1 (Oxford Instruments). The depth probed by EDS in graphite at 20 kV voltage is estimated to be 5 μm.^47^Supporting Information includes additional SEM and EDS data, collected at UWM using a Zeiss LEO 1530 at an accelerating voltage of 3 to 5 kV for SEM and at an accelerating voltage of 10 kV for EDS. At this accelerating voltage, the depth probed by EDS is less than 2 μm.

#### 2.4.2 XPS

Survey and high-resolution XPS spectra are acquired on two randomly selected points on the reference sample and on two randomly selected points per sample surface on the two exposed samples. The choice of collecting spectra on two points per sample or surface is aimed at gathering information on the variability of the XPS metrics. XPS spectra are recorded using a Thermal Scientific K-alpha spectrometer with a monochromatic Al Kα (1486.6 eV) excitation source. Beryllium safety handling procedures are described in Section 2.5. Survey XPS spectra are acquired at 0.5 eV energy step size and 1.00 eV narrow scans. High-resolution XPS spectra are recorded at a 12 kV nominal operating voltage, with a 400 μm spot size and 50 eV pass energy with 100 scans. The sampling depth for XPS spectra is between 2 and 12 nm for binding energies between 200 and 1400 eV.^48,49^ XPS depth profiling is performed on two points of the polished face of the sample exposed to liquid FLiBe (L240_P). Each depth-profiling step is composed of 2 keV monatomic Ar^+^ ions sputtering for 120 s, followed by high-resolution acquisition of C 1s, O 1s, and F 1s spectra. Nine depth-profiling steps are performed. Each step is estimated to remove a thickness corresponding to approximately 10 nm: argon sputtering size is estimated to be five times the X-ray spot size (400 μm); assuming an Ar+ current *I* = 1 μA, the flux of Ar ions on the surface is calculated as ø = *I*/*e*/*A* = 1 × 10^15^ Ar/s cm^2^, where *e* is the electron charge and *A* is the sputtering area. Considering a C–C planar bond length *l*_C–C_ = 0.142 nm,^50^ the area of a 2D carbon hexagonal cell is ; with two full atoms in each hexagon, the surface density of carbon atoms is *S*_C_ = 2/*A*_C_ = 3.85 × 10^15^ at C/cm^2^; assuming an Ar sputtering yield ξ = 1,^51^ a 120 s depth profiling step leads to the removal of *N* = 120 ξ ø/*S*_C_ = 31 monolayers of carbon atoms; considering a graphite interplanar distance of 0.335 nm,^50^ we estimate that one sputtering step removes a thickness of approximately 10 nm.

Charging effects are corrected on all spectra using the nonfunctionalized sp^2^ carbon C at 284.3 eV as an internal reference. All corrections are less than 0.2 eV, and raw XPS data is provided as Supporting Information. Peak analysis is performed using SDP v9.0 fitting software from XPS International. Recorded spectra are smoothed using 5-point Gaussian smoothing and baseline-subtracted with a Shirley baseline before peak-fitting. Fitting of the O 1s and F 1s peaks is performed using symmetric 80% Gaussian–20% Lorentzian peaks.^24,52^

#### 2.4.3 Raman Spectroscopy

Raman spectra are acquired at five randomly selected points on the reference sample and five randomly selected points per sample surface on the two exposed samples. The choice of collecting spectra on multiple points per sample or surface is aimed at gathering information on the intrasample variability of the Raman metrics. Raman spectra are recorded at Lawrence Berkeley National Laboratory (LBNL) using a Horiba LabRam HR confocal Raman microscope with a 532 nm laser source and an optical magnification of 50×. Beryllium safety handling procedures are described in Section 2.5. The slit size is set to 200 nm, and Raman spectra are collected in the 1000–3000 cm^–1^ wavenumber range. The depth probed by the laser source is estimated at 50–60 nm,^53−55^ and the sampling diameter is on the order of 2 μm.^54^ Raman spectra are fitted using Lorentzian functions on OriginPro 2021b. Crystallite parameters are estimated using the correlations provided in refs (56−58). Statistical analysis of crystallite parameters is performed using two-sample *t* tests.

### 2.5 Beryllium Safety

Gloveboxes, fume-hoods, and personal protective equipment are used to provide protection from respiratory and dermal exposure to beryllium. Beryllium contamination in the laboratory is monitored by swipes of laboratory surfaces and air monitoring in the laboratory that houses the gloveboxes. The experimental work at UWM was performed from November 2018 to June 2019 during which 29 surface swipes were analyzed. Any detection of beryllium above the detection limit of 0.025 μg/100 cm^2^ (five swipe samples with detectable Be) was addressed by cleaning and decontamination procedures. The housekeeping goal for the laboratory in which this work was performed is 0.2 μg/100 cm^2^ (the free-release limit); it was exceeded three times and was followed by cleaning of the laboratory floor and surfaces and updating procedures for moving samples between glovebox and fume-hood work. The DOE-recommended housekeeping limit of 3 μg/100 cm^2^ (10 CFR 850) was not exceeded in any of these instances. Before characterization, samples are sonicated in DI water for 2 min. Characterization of the samples, performed at UWM and UCB, from 2019 to 2023, was performed after reviewing handling protocols with instrument managers. Personal protective equipment (PPE) used during characterization include double-layered disposable gloves (changed at every contact with the sample) and lab coats. Sample stubs and stages used during characterization are wiped clean with water or ethanol after each use.

## 3 Results

SEM, XPS, and Raman results are presented for the polished surface of the samples (Ref_P, L240_P, and G240_P). Characterization results for the as-machined surfaces (Ref_M, L240_M, and G240_M) are included as Supporting Information and discussed in Section 4.2.2.

### 3.1 SEM/EDS Analysis

**Figure 2:**
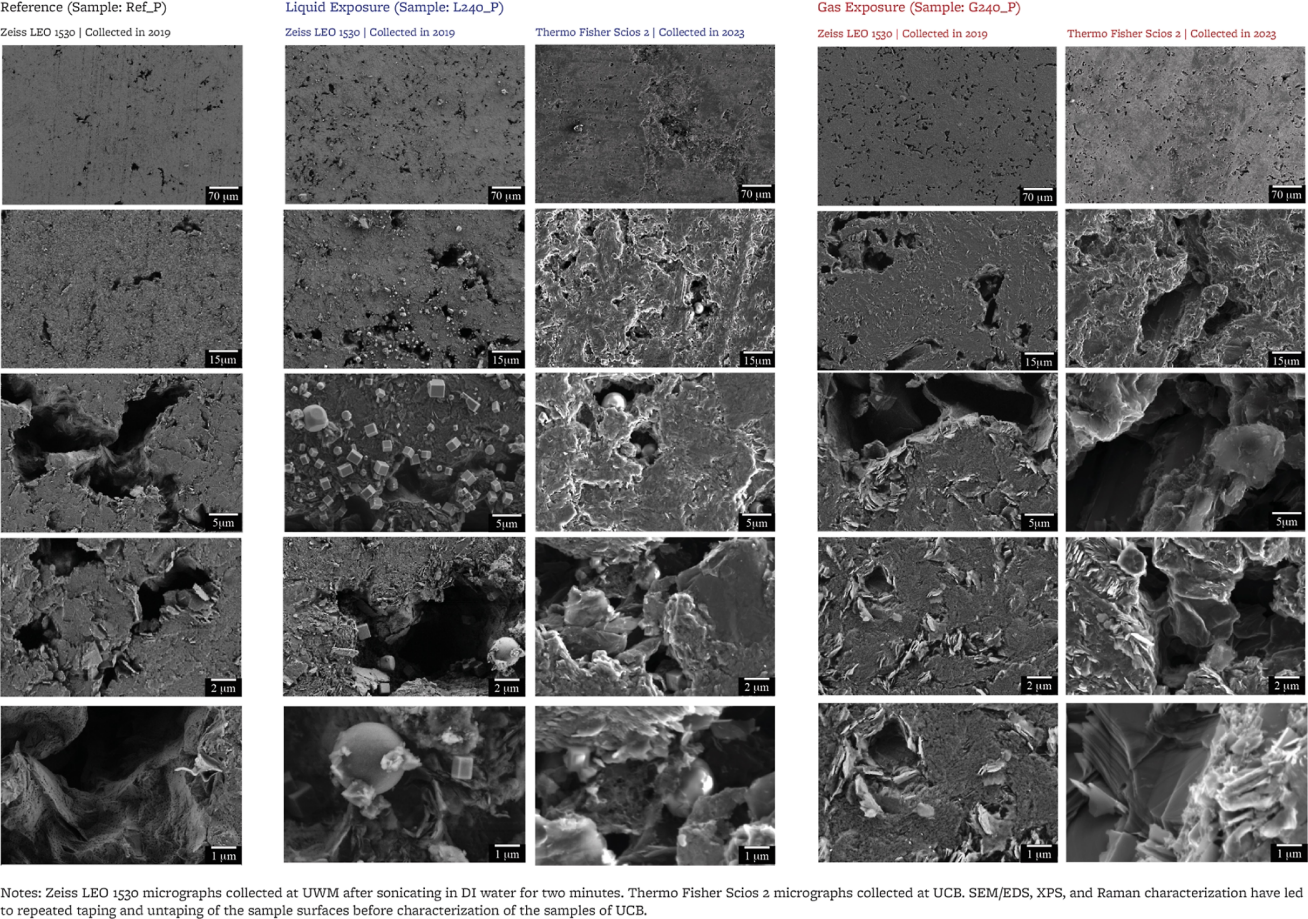
SEM micrographs of the polished surfaces of reference and exposed samples. For each sample, micrographs shown here are collected at up to two locations and may not be representative of the overall sample. Additional images provided as Supporting Information.

**Figure 3:**
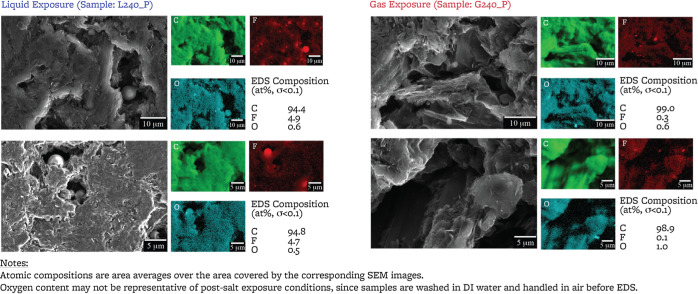
EDS collected on the polished surfaces of exposed samples. Scans are acquired at locations where the spherical particles are observed and may not be representative of the overall sample. Additional EDS maps and point spectra provided as Supporting Information.

Figure 2 shows SEM micrographs for the polished surface of the three samples (sampling depth ∼3 m^47^). Flakes of few microns size are observed on all samples (Figure 3). Spheres of 1 to 4 μm diameter are observed inside of the pores of the liquid FLiBe-exposed sample, and spheres of 1 to 2 μm diameter are observed in the pores of the cover gas-exposed sample. In the area surrounding the spheres, EDS (sampling depth ∼5 μm^47^) shows signals predominantly from C, F, and O (Figure 3), and the spheres only indicate a signal from F and little or no signal from C; Li and Be are not detectable by EDS, thus the spheres observed here may be either BeF_2_ and/or LiF, and the surrounding area may contain C–F compounds.

We acknowledge that slightly different pre-baking conditions were used for the 12 h and the 240 h salt exposure tests, though we consider the differences not to be of significance to the discussion of CF formation. The prebaking for the 240 h salt exposure was done in vacuum at 1500 °C for 12 h, with samples being supported by an alumina boat, giving a baking mass loss of 0.08(2)% and surface oxygen content of 5.4(1.4)% (from XPS); we consider these conditions comparable with the prebaking conditions of the 12 h exposed sample, pre-baked in a stainless steel vessel in argon gas at 1000 °C for 3 h, for which a 0.029(8)% mass loss was recorded and a surface oxygen content of 11% (from XPS). The two sets of control samples have similar Raman spectra, with similar *I*_D_/*I*_G_, and slightly narrower D and G peaks for the sample pre-baked at 1500 °C. We note that submicron-sized pits are visible on all three samples pre-baked at 1500 °C (control, salt-exposed, and gas-exposed), which we postulate to have been caused by the use of the alumina boat while pre-baking; appearances of a pitted surface with holes of tens of micron diameter and of micron-sized flakes were previously observed upon oxidation of samples made of nuclear graphite grades IG-110 and NBG-18 in dry air at 1100 °C and above;^59^ in our case, the oxygen partial pressure was much lower, and oxidation was minimal, as illustrated by low mass loss and Raman and XPS comparison of the two sets of control samples.

### 3.2 XPS Surface Analysis

**Figure 4:**
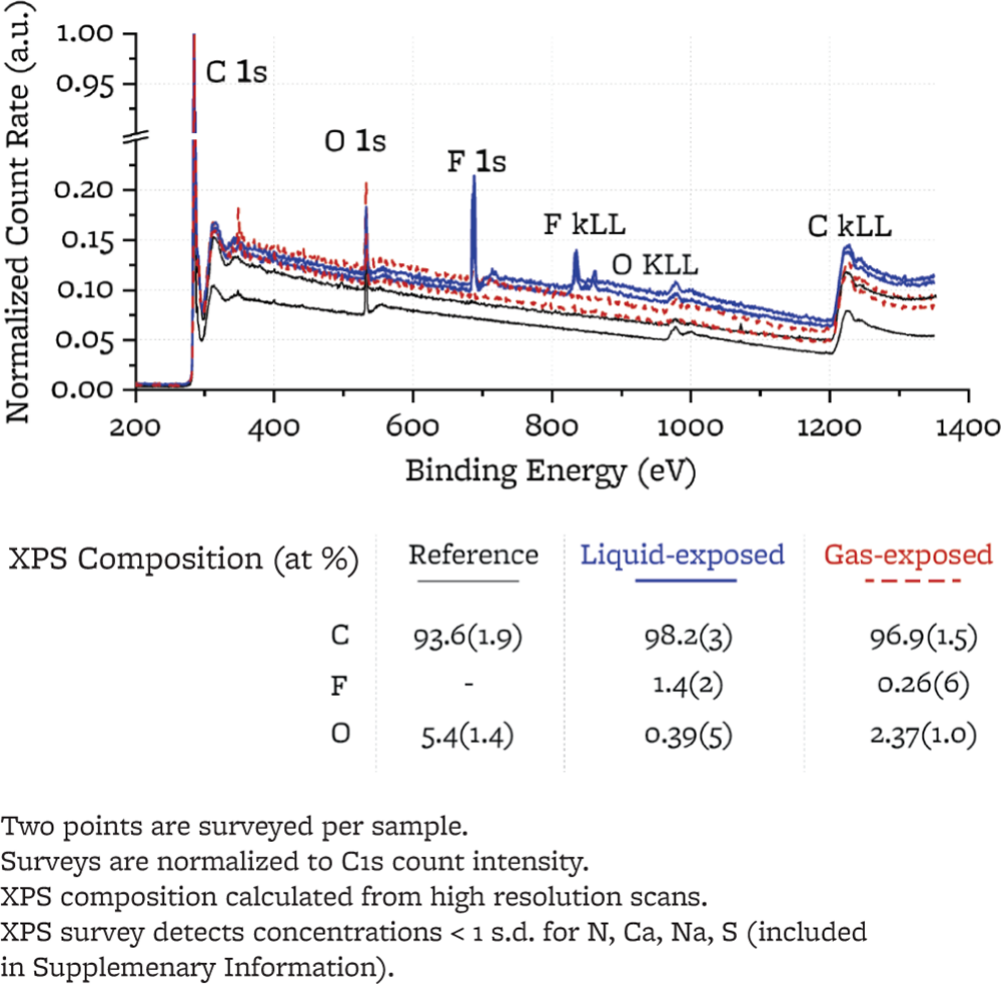
XPS survey of the polished surfaces of the reference and exposed samples.

XPS spectra (sampling depth: 2–12 nm,^48,49^ sampling diameter: 400 μm) are collected at two locations on each sample to investigate the chemical composition on the surface of the graphite samples. The survey scans (Figure 4) are consistent with published XPS spectra for samples of IG-110 grade graphite,^15,59−62^ which show a strong C 1s peak and a smaller O 1s peak.^15,59,60,62^ The presence of F 1s and F KLL peaks on the exposed samples indicates the appearance of fluorine species on graphite surface upon exposure to both liquid FLiBe and the cover gas above it. The XPS survey analysis does not reveal Be or Li signals from the sample surface. The lack of peaks in the region associated with Li (BE ∼ 55 eV) and Be (∼115 eV) may be caused by the very low sensitivity factors of Li and Be (SF_Li_ = 0.08, SF_Be_ = 0.2, compared to SF_C_ = 1, as provided by SDP v9.0). High-resolution XPS scans are acquired around the C 1s, O 1s, and F 1s peaks but are not acquired in the regions associated with Li and Be as their peaks are not observed in the surveys.

#### 3.2.1 C 1s Peaks

High-resolution C 1s peaks for two points on each sample are shown in Table 4. All spectra are normalized to the same maximum intensity and the same peak maximum location of 284.3 eV, and less than 0.2 eV correction was needed for each spectrum. The C 1s peak is fitted with one asymmetric subpeak (peak C sp^2^) and seven symmetric subpeaks C_1_ to C_7_. Three of the symmetric subpeaks (C_1_, C_2_, and C_7_) are assigned to carbon atoms. The remaining four subpeaks (C_3_, C_4_, C_5_, and C_6_) are in the region 285.5–289, which has been previously associated with oxygen-containing carbon functional groups (OC) and fluorine-containing carbon functional groups (FC).^24−26,34,63^ The high, intrasample, point-to-point variability of the C 1s subpeak areas makes it difficult to identify from the C 1s spectra alone a difference in the sp^3^, point defects, OC, and FC content across samples, and analysis of F 1s, O 1s, and C KLL peaks is necessary to draw quantitative conclusions, and an example self-consistency check between C 1s, O 1s, and F 1s spectra is given in Supporting Information.

#### 3.2.2 C KLL Peak and *D*-Parameter

**Figure 6:**
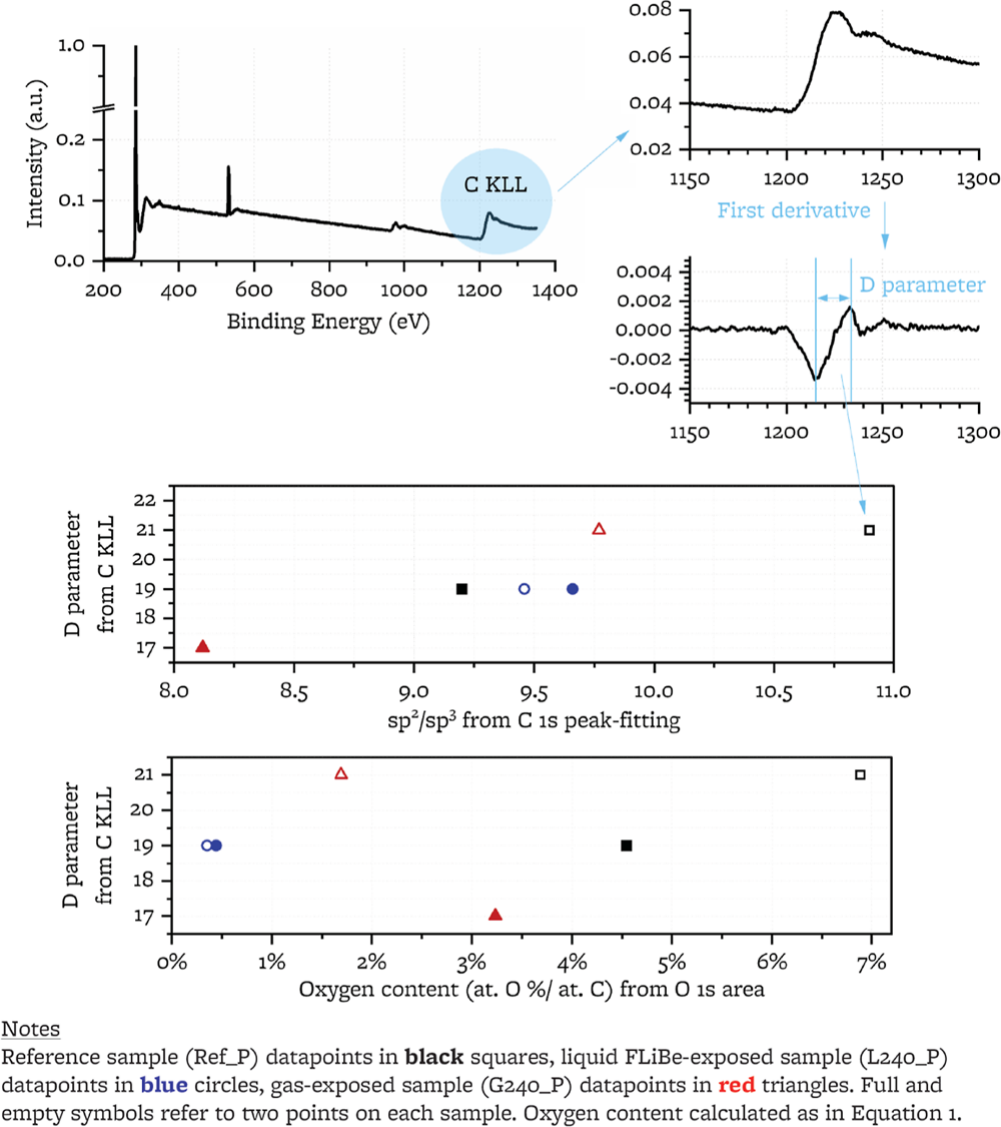
Example of *D* parameter calculation from C KLL peak and correlation of *D* parameter with sp^2^/sp^3^ ratio from C 1s peak and with oxygen content from O 1s area.

The ratio of sp^2^ to sp^3^ bound carbon atoms can be estimated by peak-fitting C 1s peaks.^52,64,67,73−75^ However, due to the presence of C–F and C–O bonds, peak fitting of the C 1s peaks on these samples can be ambiguous (discussed in Section 4.1.1). Instead, the *D* parameter (i.e., the difference between the maxima and minima of the first derivative of the C KLL spectra^67,76^) has been shown to correlate linearly with the sp^2^ content^76^ and is an independent metric for sp^3^ content that removes the ambiguity from C 1s peak-fitting. *D*-parameters are calculated after 3-point adjacent averaging for all C KLL peaks^48^ (Figure 6). Reference (48) reports that the *D*-parameter is influenced by oxygen content; plotting the *D*-parameter against the oxygen content (Figure 6), we do not observe a correlation for the present samples with their respective oxygen content (Figure 6). The calculated *D*-parameters correlate linearly with the sp^2^/sp^3^ ratios from C 1s peak fitting (Figure 6), and both show large intrasample variability (e.g., 4 units change across the two points of the sample exposed to the liquid salt). This variability may reflect heterogeneity of sp^2^ and sp^3^ carbon content across locations of the sample (e.g., filler, binder, pore edge). We conclude that a change in sp^3^ content does not take place with exposure.

#### 3.2.3 O 1s Peaks

High-resolution O 1s peaks for two points on each sample are fitted with four symmetric subpeaks, O_1_ to O_4_, following the OC peak assignment in ref (65) (Table 5). OC content is quantified by Ω
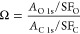
1where SF_O_ and SF_C_ are oxygen and carbon sensitivity factors, respectively, (SF_O_ = 2.9, SF_C_ = 1 provided by SDP v9.0). Ω decreases by a factor of 2 upon cover-gas exposure and by a factor of 10 upon salt exposure. O_4_ (O–C=O) is the only subpeak that may be preferentially decreased upon exposure to the cover gas; the rest of the peaks remain in proportions similar to the reference sample, i.e., the O 1s changes in magnitude but not in shape upon salt and cover gas exposure.

**Table 5:**
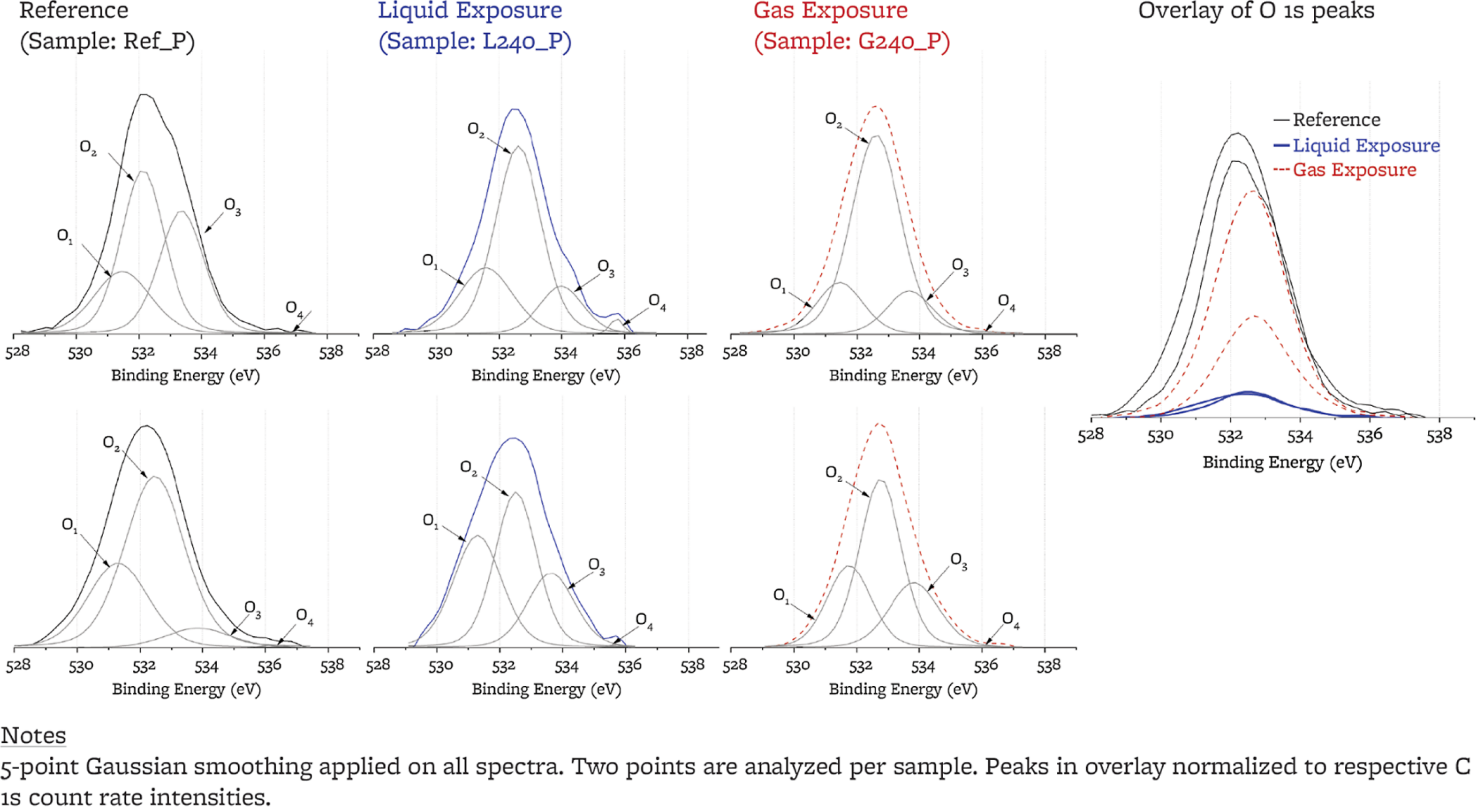
XPS O 1s Peak Fitting for Spectra Collected on the Polished Surfaces of Reference and Exposed Samples^a^

O 1s sub-peak	BE (eV), normalized to 284.3 eV C 1s peak|FWHM (eV)						O 1s sub-peak interpretation	
	area (%), relative to the total O 1s peak area							
	reference (Ref_P)		liquid exposure (L240_P)		gas exposure (G240_P)			
	point 1	point 2	point 1	point 2	point 1	point 2		
O_1_	531.5|2.1	531.4|2.2	531.6|2.0	531.3|1.8	531.5|1.7	531.8|1.6	C=O	(65)
	21.8%	30.3%	24.5%	35.0%	16.9%	25.8%		
O_2_	532.1|1.6	532.5|2.3	532.6|1.7	532.5|1.6	532.6|1.9	532.8|1.6	C–O in epoxy structure	(65)
	43.7%	61.7%	59.7%	42.3%	69.1%	50.8%		
O_3_	533.4|1.7	533.9|2.4	534.0|1.6	533.6|1.8	533.7|1.7	533.8|1.9	C–O in ether structure	(65)
	34.2%	7.6%	14.4%	22.7%	14.0%	23.4%		
O_4_	536.2|1.1	536.3|1.0	535.7|0.5	535.7|0.5	535.8|0.5	536.6|0.5	O–C=O	(65)
	0.3%	0.4%	1.4%	0.3%	0.1%	0.1%		
Ω	4.5%	6.9%	0.4%	0.4%	3.2%	1.7%	at. % of carbon-bound oxygen relative to total number of carbon atoms	

a Rows are ordered by type of peak assignment. Area percentages are relative to the total O 1s peak area for each sample.

#### 3.2.4 F 1s Peaks

High-resolution F 1s peaks for two points on each sample are fitted with five symmetric subpeaks, F_1_ to F_5_ (Table 6). Low-energy peaks are assigned to the salt species LiF, LiBeF_3_, and BeF_2_ and higher energy peaks are attributed to two types of C–F bonds. When they were first observed, several authors referred to these two types of bonds as semi-ionic (F_3_)^24,26,33,63,71,77^ and covalent (F_4_)^26,63,77,78^ C–F bonds, implying that semi-ionic bonding does not cause a change of sp^2^ hybridization, while covalent bonding leads to local sp^3^ carbon hybridization. More recently, in the light of nuclear magnetic resonance (NMR) and X-ray absorption near edge structure (XANES), other authors have argued against the existence of a semi-ionic bond, rather suggesting that both bonds are covalent, although they may have a different C–F bond length (0.140 vs 0.136 nm) and hence energy.^71,79−82^ For simplicity of notation and consistency with graphite fluorination works that include XPS analysis, we refer to the two types of bonds as semi-ionic and covalent in this paper, but we recognize that further studies through XANES and NMR would be required to ascertain the nature of the bonds that we observe.

**Table 6:**
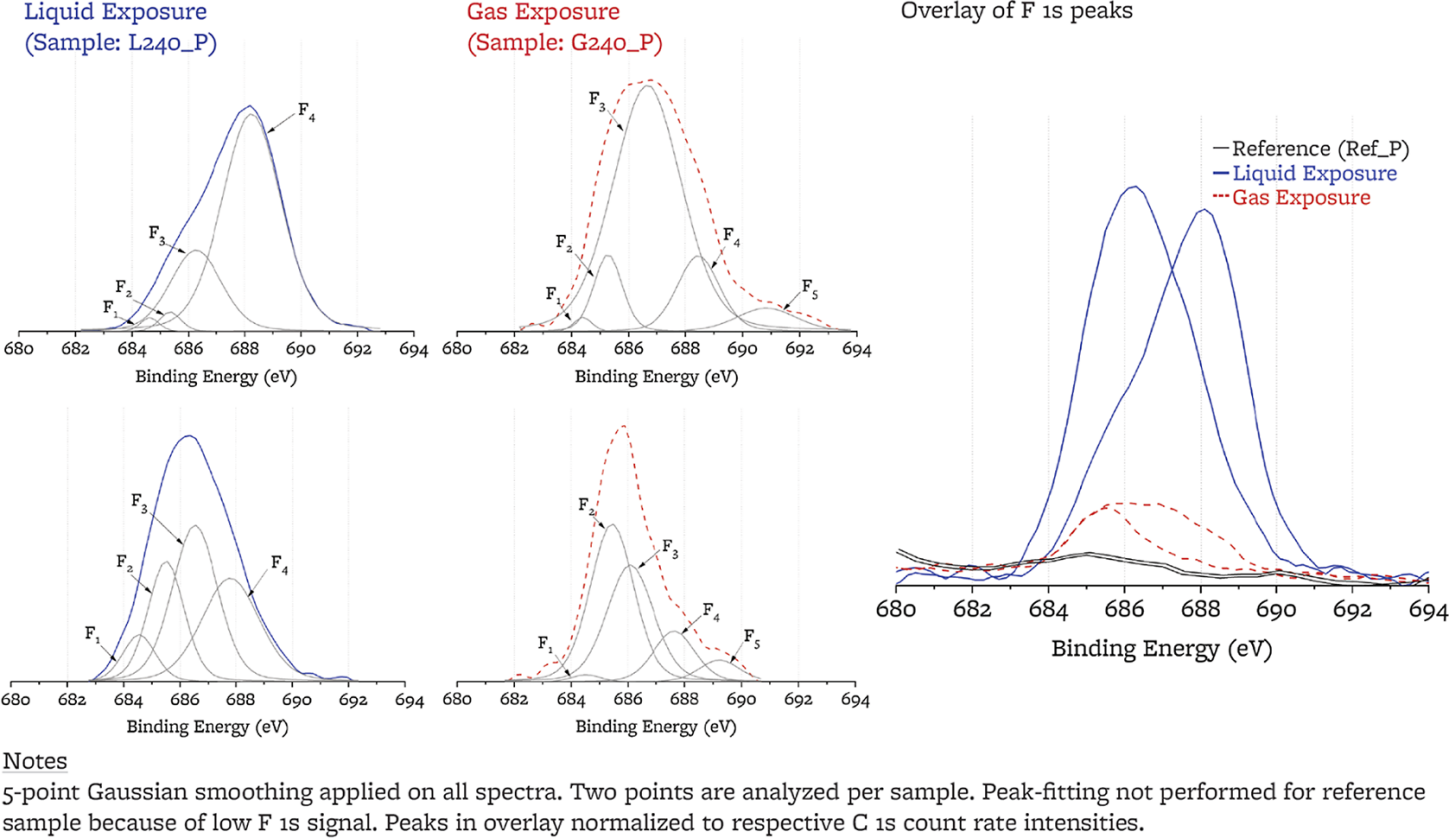
XPS F 1s Peak Fitting for Spectra Collected on the Polished Surfaces of Exposed Samples^a^

F 1s sub-peak	BE (eV), normalized to 284.3 eV C 1s peak|FWHM (eV)				F 1s sub-peak interpretation	
	area (%), relative to the total F 1s peak area					
	liquid exposure (L240_P)		gas exposure (G240_P)			
	point 1	point 2	point 1	point 2		
F_1_	684.6|0.8	684.5|1.4	684.5|0.7	684.5|1.2	LiF	(85−86,87,88)
	1.4%	8.1%	1.1%	1.3%	or LiBeF_3_	hypothesized
F_2_	685.3|0.9	685.5|1.5	685.4|1.2	685.4|1.8	BeF_2_	(89,90)
	2.3%	22.9%	9.1%	44.3%		
F_3_	686.3|2.2	686.5|1.9	686.8|2.9	686.1|1.9	semi-ionic C–F bonds	(24,26,33,63,71,77)
	23.6%	37.5%	70.8%	35.2%		
F_4_	688.2|2.5	687.8|2.4	688.6|1.7	687.6|1.7	covalent C–F bonds	(26,63,77,78)
	72.6%	31.6%	13.3%	13.5%		
F_5_			690.9|2.5	689.2|1.7	covalent C–F bonds in CF_2_ and CF_3_ groups	(26)
			5.8%	5.7%		
F_2_/F_1_	1.7	2.8	8.5	33.2	liquid: expect 1 = (2 × BeF_2_)/LiF	
					vapor: expect 8 = (2 × BeF_2_)/(3 × LiBeF_3_) at 700 °C^84^ --------------------------------or expect 10^7^ = (2 × BeF_2_)/LiF at 700 °C^84^	
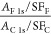	1.6%	1.2%	0.3%	0.2%	F/C (at. %)	
Φ	1.6%	0.8%	0.3%	0.1%	degree of fluorination, F_CF_/C (at. %)	
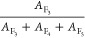	24.5%	54.2%	78.8%	64.8%	F_semi-ionic_/F_CF_ (at. %)	

a Rows are ordered by type of peak assignment.

In the liquid-exposed sample, the prevalence of C–F covalent bonds is larger; the ratio of covalent to semi-ionic C–F bonds is 1.5:1 in the liquid-exposed and 1:2.5 (0.4:1) in the gas-exposed. Both F_3_ and F_4_ have a large FWHM (>1.7 eV), suggesting that they may be in turn composed of subpeaks associated with different bonds. An additional subpeak, F_5_, is required for the fitting of the spectra of the sample exposed to gas above the salt, and it is attributed to covalent C–F bonds in CF_2_ and CF_3_ groups.^26^

The total amount of carbon-bound fluorine atoms (F_CF_) relative to the total number of carbon atoms is quantified by ϕ, the degree of fluorination
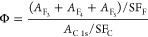
2where SF_F_ is the fluorine sensitivity factor (SF_F_ = 4.43 provided by SDP v9.0). Averaging over the two spectra of each sample, ϕ_liq_ = 1.2(5)% and ϕ_gas_ = 0.2(1)% (6 times lower with cover gas exposure).

The relative abundance of BeF_2_ to LiF can be computed from the area ratio of subpeaks F_1_ and F_2_, with correction for the stoichiometric ratio of fluorine atoms bound to Li and Be. In liquid FLiBe, BeF_2_/LiF is nominally 0.5, and the corresponding F_2_/F_1_ area ratio would be 1. In the gas phase in equilibrium with FLiBe at 700 °C, the equilibrium BeF_2_/LiF ratio is 10^7^ at 700 °C, and the BeF_2_/LiBeF_3_ vapor phase ratio is 11 at 700 °C and 16 at 460 °C. If F_1_ is attributable to LiBeF_3_, then the corresponding F_2_/F_1_ ratio for the condensed (quenched) gas phase would be 8 at 700 °C and 11 at 460 °C (correcting for the fluorine stoichiometry).^83,84^ For the liquid-exposed sample, the observed F_2_/F_1_ ratio is 2.2(8); this value being above unity indicates that some amount of gas transport into the surface porosity may be occurring. For the gas-exposed sample, the observed F_2_/F_1_ ratio is 20(17), much higher than for the liquid-exposed sample, reinforcing the correct attribution of the F_1_ and F_2_ subpeaks to halide salts.

### 3.3 Raman Analysis

**Figure 9:**
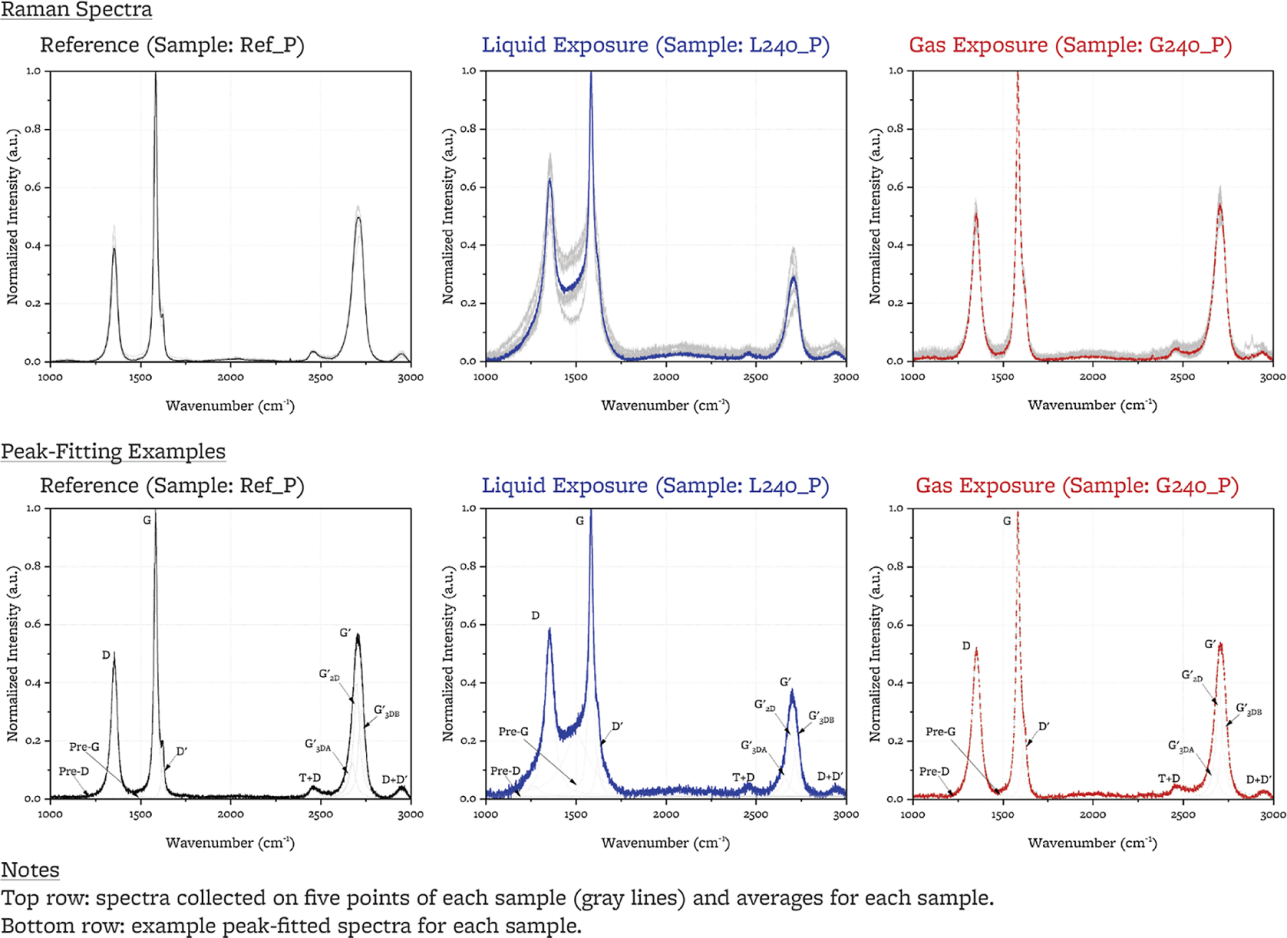
Raman spectra collected on the polished surfaces of the reference and exposed samples.

Raman spectra (sampling depth: approximately 50–60 nm,^53−55^ sampling diameter: approximately 2 μm^54^ with a 532 nm laser source) are collected at five points on each sample (Figure 9). The location of the peaks composing the spectra is consistent with previous results for nuclear graphite. Raman spectra for IG-110 were included in refs (15,46, and 91) and show narrow D, G, and D′ bands at approximately 1350, 1580, and 1610 cm^–1^ wavenumbers in the first order spectrum and T + D, G′, and D′ + D band at approximately 2450, 2700, and 2950 cm^–1^ wavenumbers in the second order spectrum, respectively.

Upon exposure, multiple features of the Raman spectra are shown to change: the intensity of the D band and full width half-maximum (FWHM) of the G band increase, the intensity between the D and the G bands does not fall below 0.1, leading to the appearance of a bridge, and the intensity of the G′ band decreases. Peak-parameters of the D, G, and G′ bands are used to estimate graphite crystallite parameters *L*_a_ and *L*_c_, according to the correlations in refs (56−58) and as shown in previous studies that used Raman spectroscopy to characterize graphite^92^ (Table 7). We observe an increase of the *I*(D)/*I*(G) ratio and a broadening of the FWHM of the G band, which correlate to a statistically significant decrease of the basal crystallite parameter *L*_a_(56−58) (50% decrease for salt-exposed and 40% decrease for cover-gas-exposed). The intensity of the G′ band decreases with exposure, but the peak decomposition between the G′2D and the two G′3D peaks does not change significantly for either sample, yielding a degree of stacking order *R* and a crystallite parameter *L*_c_ statistically unchanged with exposure (Table 7). Overall, this suggests that exposures to the liquid salt and the gas above yield the same type of microstructural changes, and these changes are more pronounced upon liquid exposure.

**Table 7:** Raman Figures of Merit and Crystallite Microstructural Parameters (Calculated from Raman Spectra in Figure 9)^a^

	reference (Ref_P)	liquid exposure (L240_P)	gas exposure (G240_P)
**I(D)/I(G)**	**0.41(6)**	**0.68(12)**	**0.54(4)**
≠ Ref_P (*p*-value)		0.001	0.002
G240_P ≠ L240_P (*p*-value)			0.03
**FWHM(D) (cm^–1^)**	**36(1)**	**67(7)**	**50(2)**
≠ Ref_P (*p*-value)		<0.001	<0.001
G240_P ≠ L240_P (*p*-value)			0.001
**A(G′) (% of total spectrum area)**	**43(1)%**	**14(5)%**	**35(1)%**
≠ Ref_P (*p*-value)		<0.001	<0.001
G240_P ≠ L240_P (*p*-value)			<0.001
degree of stacking order R^58^ from G′ peak decomposition	47(8)%	41(10)%	41(6)%
≠ Ref_P (*p*-value)		0.34	0.19
G240_P ≠ L240_P (*p*-value)			0.88
c (nm)^58^ from G′ peak decomposition	0.6768(9)	0.6774(11)	0.6775(7)
≠ Ref_P (*p*-value)		0.34	0.19
G240_P ≠ L240_P (*p*-value)			0.88
**L_a_ (nm)^56^ from D/G area ratios**	**27(4)**	**13(2)**	**18(1)**
≠ Ref_P (*p*-value)		<0.001	<0.001
G240_P ≠ L240_P (*p*-value)			<0.001
**L_a_ (nm)^57^ from FWHM(G)**	**68(10)**	**30(7)**	**43(6)**
≠ Ref_P (*p*-value)		<0.001	<0.001
G240_P ≠ L240_P (*p*-value)			0.014
L_c_ (nm)^58^ from FWHM(G)	27(3)	26(2)	26(1)
≠ Ref_P (*p*-value)		0.37	0.18
G240_P ≠ L240_P (*p*-value)			0.76

a Values in bold indicate a significant difference (*p* < 0.05). Raman sampling depth: ∼120 nm,^54^ sampling diameter: ∼1 μm^46^

### 3.4 Effect of Exposure Duration

To investigate the time-evolution of graphite exposed to molten FLiBe, the 240 h exposure from this study is compared with the 12 h exposure from ref (15). XPS and Raman spectra from ref (15) are reanalyzed to be methodologically consistent with the new data presented here, since some of the peak fitting parameters used in ref (15) (e.g., number and shape of XPS and Raman peaks, Raman constraints) differ from those used here. All reanalyzed results are included as Supporting Information. Table 8 provides a summary of the XPS and Raman characterization of the 12 and 240 h samples.

**Table 8:** Summary of Comparative Metrics of Graphite Surface Modifications upon Exposure to FLiBe at 700 °C

technique	depth of sampling (nm)	metric	reference (Ref_P)	12 h liquid exposure (L12_P)^15^	240 h liquid exposure (L240_P)	240 h gas exposure (L240_P)
**XPS**, sampling diameter = 400 μm^a^			*n* = 2	*n* = 1	*n* = 2	*n* = 2
survey	2–12	O (at. %)	5.4(1.4)	1.0	0.39(5)	2.37(11)
		F (at. %)		0.2	1.4(2)	0.27(7)
		C (at. %)	93.6(1.9)	98.2	98.2(3)	96.9(1.5)
O 1s	8	Ω =C–O/C (at. %)	5.7(1.7)	1.0	0.40(6)	2.5(1.1)
F 1s	7	F_2_/F_1_ (subpeak area ratio)		2.6	2.2(8)	20(18)
		F/C (at. %)		0.3	1.4(2)	0.27(7)
		**ϕ C–F/C** (**at. %)**		**<0.1**	**1.2(5)**	**0.20(12)**
		% SI =semi-ionic C-F/C–F (at. %)		74	39(21)	72(10)
		ϕ_semi__-__ionic_ = semi-ionicC–F/C (at. %)		<0.07	0.5(3)	0.14(8)
		ϕ_covalent_ = covalentC–F/C (at. %)		<0.03	0.7(3)	0.06(8)
C 1s	10	sp^2^/sp^3^ from C 1s	10.1(1.2)	9.6	9.7(2)	9.0(1.2)
C KLL	3	*D* parameter (indicative of sp^2^/sp^3^)	20 (1)	19	19 (−)	19 (2)
Raman, sampling diameter = 2 μm^54^			*n* = 5	*n* = 5	*n* = 5	*n* = 5
	50–60^53−55^	*I*(D)/*I*(G)	0.41(6)	0.60(17)	0.68(12)	0.54(4)
		FWHM(D)	36.4(1.0)	81(25)	66(7)	50(2)
		FWHM(G)	20.4(0.8)	29(5)	29(3)	24.1(1.7)
		*A*(G′)	43.2(1.0)	14(2)	15(5)	35.1(1.2)
		degree of graphitization (%)^58^	47(8)	33(16)	41(10)	41(6)
		*c* (nm)^58^	0.6768(9)	0.6784(18)	0.6774(11)	0.6775(7)
		*L*_a_ (nm)^56^	27(4)	13(3)	13(2)	17.6(6)
		*L*_c_ (nm)^58^	27(3)	25(4)	26(2)	25.6(1.4)
data analysis^b^						
semi-ionic C–F		C_bulk_/C_total_ (%)	96.6(5)	93.0(1.6)	93.0(1.1)	94.8(1.8)
		semi-ionic C–F/C_bulk_ (%)		0.08(1)	0.5(3)	0.15(2)
covalent C–F		C_edge_/C_total_ (%)	3.4(5)	7.0(1.6)	7.0(1.1)	5.2(1.8)
		covalent C–F/C_edge_ (%)^c^		0.37(9)	10.4(3.9)	1.1(5)
		C–O/C_edge_ (%)^c^	160(50)	14(3)	5.7(1.2)	50(30)
		(covalent C–F + C–O)/C_edge_ (%)^c^	160(50)	15(3)	16(4)	50(30)
		ϕ_covalent_ + Ω (at. % of C)	5.7	1.0	1.1	2.56

a XPS^48,49^ sampling depth defined as the depth from which 99% of the signal is originated.

b *C*_bulk_ = *C*_total_ – *C*_edge_ and *C*_edge_/*C*_total_ = (0.15 nm^3^)/(*c* × *a* × *L*_a_) from eq 15 of ref (44), with *a* = 0.2465 nm from XRD from ref (46) and *c* and *L*_a_ from Raman.

c Since one or multiple F atoms can be bound to a common C atom, the % C–F and % C–O occupancy of the edge atoms on graphite crystallite surfaces can be smaller than the covalent C–F/*C*_edge_ ratio.

### 3.5 Effect of Graphite Surface Finish

To test for an effect of the graphite surface finish, data was also collected on the as-machined (unpolished) surfaces of the samples (preparation described in Table 3). Summary data is included in Table 9. Data for all samples is available as Supporting Information. The observations discussed above for gas vs liquid phase exposure remain the same across this broader set of samples.

**Table 9:** Parameters Impacting Surface Fluorination of Graphite by Exposure to Molten FLiBe at 700 °C

		exposure duration		exposure type		graphite surface finish	
		12 h	240 h	liquid FLiBe	cover gas	polished	machined
samples:		L12_P	L240_P	L240_PL240_M	G240_PG240_M	L240_PG240_P	L240_MG240_M
XPS (2–12 nm sampling depth, 400 μm sampling diameter)	points analyzed per sample	1	2	2	2	2	2
	ΔΩ (vs ref sample)	4.76%	5.32(6)%	5.32(6)%^a^	3.3(1.1)%^a^	4.3(1.3)%	no reference
	F_2_/F_1_ (salt specis, e.g. 2BeF_2_/LiF)	0.8	1.0(4)	0.7(4)	0.3(2)	0.6(5)	0.47(12)
	ϕ = F_CF_/C	<0.1%	1.2(5)%	0.9(5)%	0.26(9)%	0.7(6)%	0.40(17)%
	% SI = ϕ_SI_/ϕ	76%	39(21)%	27(19)%	42(19)%	56(23)%	30(23)%
	ϕ_SI_ = F_semi-ionic CF_/C	0.04%	0.42(6)%	0.2(2)%	0.15(8)%	0.29(17)%	0.10(7)%
	ϕ_cov._ = F_covalent CF_/C	0.01%	0.8(6)%	0.6(4)%	0.11(7)%	0.4(5)%	0.3(2)%
	observations	higher ϕ, ϕ_SI_, ϕ_cov_ and lower %SI with longer exposure time		lower % SI with liquid vs gas exposure		large standard deviations prevent comparison	
Raman (50–60 nm sampling depth, 2 μm sampling diameter)	points analyzed per sample	5	5	5	5	5	5
	*I*(D)/*I*(G)	0.60(17)	0.68(12)	0.53(15)	0.43(8)	0.68(12)	0.35(11)
	FWHM(D)	81(25)	66(7)	56(10)	49(8)	58(7)	47(10)
	FWHM(G)	29(5)	29(3)	26(4)	22(2)	27(4)	22(2)
	*A*(G′)	14(2)	15(5)	28(5)	39(5)	25(5)	42(5)
	degree of graphitization (%)	33(16)	41(10)	43(12)%	44(15)%	41(11)	45(15)
	*c* (nm)^58^	0.6784(18)	0.6774(11)	0.6773(13)	0.6772(17)	0.6775(12)	0.6770(17)
	*L*_a_ (nm)^56^	13(3)	13(2)	20(5)	23(11)	15(2)	28(12)
	*L*_c_ (nm)^58^	25(4)	26(2)	26(3)	27(4)	26(3)	27(4)
	observations	surface microstructural changes appear insensitive to exposure time		more pronounced surface microstructural changes with liquid vs gas exposure		more pronounced surface microstructural changes wih polished vs machined graphite	

a Calculated only on polished samples, as machined reference not available.

### 3.6 XPS Depth Profiling

**Figure 10:**
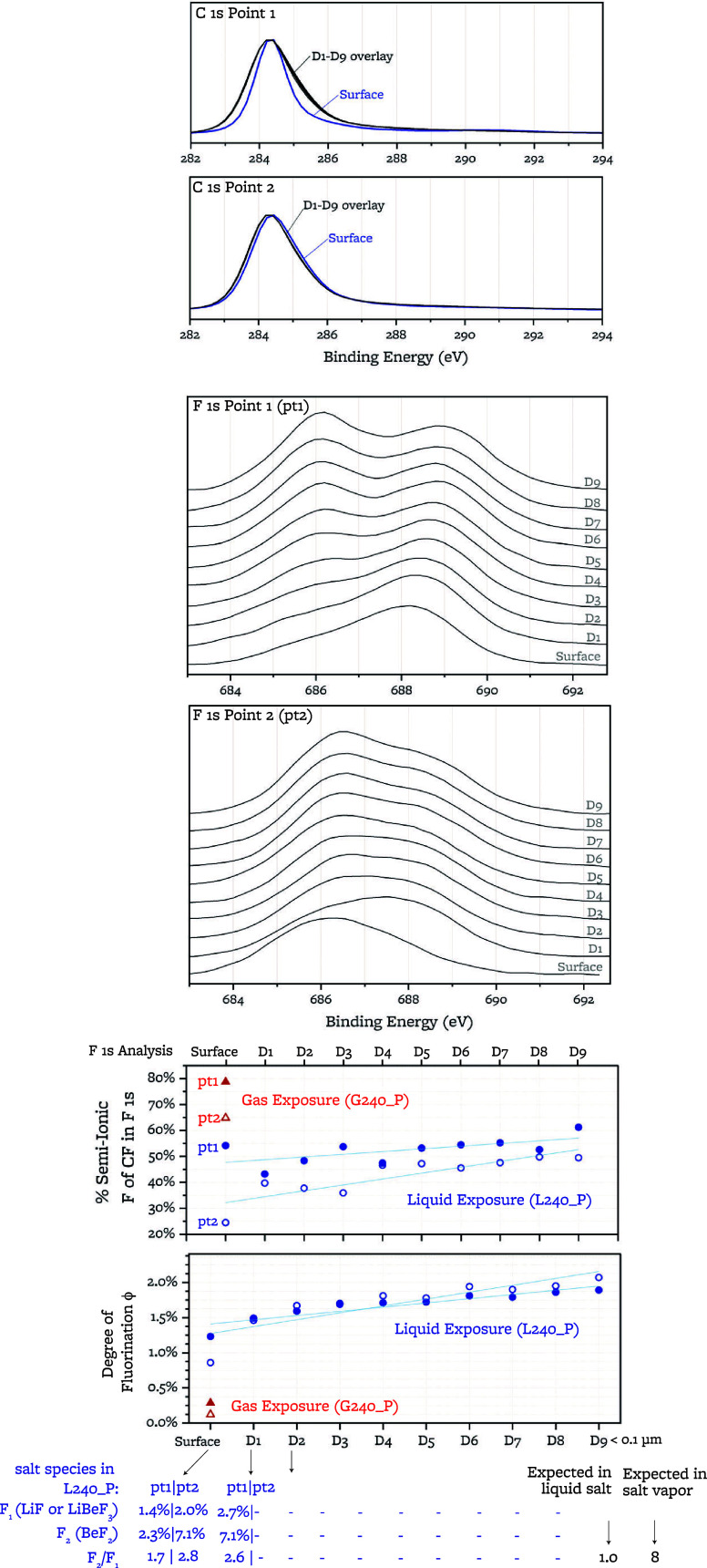
XPS depth profiling of the polished surface of the sample exposed to liquid FLiBe (L240_P).

For the liquid-salt-exposed sample, argon ion sputtering is used to acquire depth profiling for the F 1s and C 1s XPS peaks at ten depths and two points on the sample (Figure 10). We estimate that depth D_9_ is at less than 100 nm. Full peak-fitting parameters are included as Supporting Information. The C 1s peaks do not display differences with increasing depth. The F1 s peaks for both points show an increase of the fraction of C–F bonds that are semi-ionic with depth (40% at surface, 55% at D_9_) and an increase in the degree of fluorination ϕ with depth (1.2(5)% at surface, 2.0(1)% at D_9_). Halide salt species are no longer present beyond the D_1_ sputtering step, and the F_1_/F_2_ ratio remains characteristic of liquid FLiBe (and not FLiBe vapor space) at step D_1_; this would indicate that for the <1 μm depth probed, vapor-space transport for halide salt species is not of relevance to the salt-exposed sample.

### 3.7 GDMS Depth Profiling

**Figure 11:**
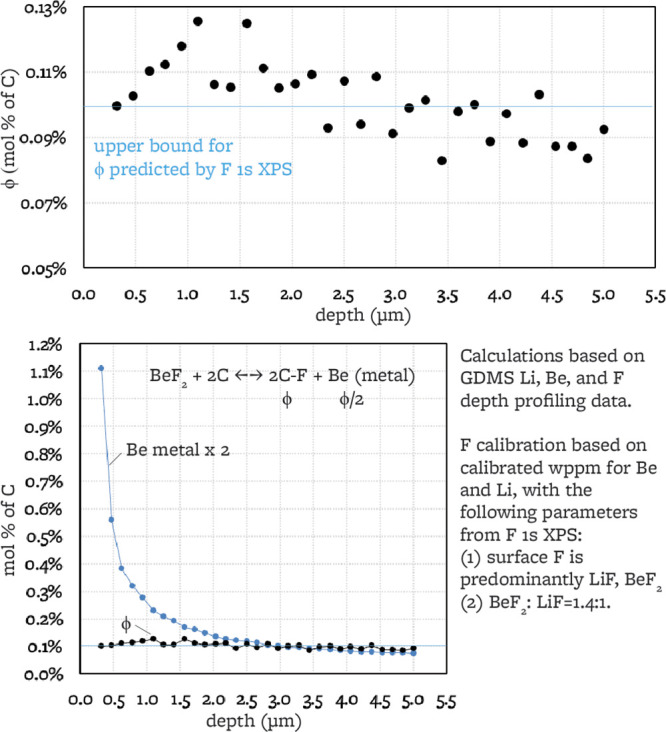
ϕ and Be metal depth profiling calculated from GDMS data for sample exposed to liquid FLiBe for 12 h (L12_P).^15^

No concentration standard was available for the fluorine data from GDMS depth profiling for the 12 h liquid FLiBe exposure sample,^15^ and only values relative to the surface were reported. With the present XPS F 1s results that identify the ratio of LiF/BeF_2_ and the ratio of C–F/(LiF + BeF_2_) at the surface, a quantitative analysis of Be metal and C–F vs depth can now be generated (Figure 11). Using the Li surface concentration determined by GDMS, the surface F concentration can be calculated and used to scale the F counts at all depths (eqs 3 and 4).
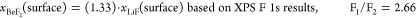
3
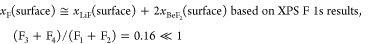
4

From the Li concentration vs depth and the now normalized F concentration vs depth, the C–F concentration versus depth is calculated as in eq 5.
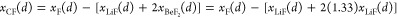
5

From the Be concentration vs depth measured by GDMS and Li and F concentration, the Be metal concentration versus depth is calculated as in eq 6
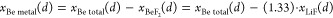
6

We remarkably find close agreement with the ϕ < 0.1% value determined from XPS F 1s for C–F surface content, with the only information used from XPS being subpeak ratios LiF/BeF_2_ and C–F/(LiF + BeF_2_). Also, remarkably, the surface Li concentration measured from GDMS is 0.096 at % Li/C, and from XPS F 1s it is 0.099 at % Li/C, showing agreement between two independent measurements.

The degree of fluorination ϕ increases with depth up to 1 μm (with a slope of 0.15%/μm). A similar observation was noted from XPS F 1s depth profiling up to 0.1 μm for the 240 h liquid-exposed sample (in this case with a slope of 8%/μm). Past 1 μm of depth in the 12 h exposed sample, ϕ begins to decrease and to exhibit more scatter.

We note that the surface Be metal concentration is much higher than the C–F concentration, and this concentration decreases much more rapidly than the C–F concentration vs depth. If we assume that all of the Be metal concentration is a consequence of the reaction in eq 8, by integrating the total content of Be metal, we deduce a C–F presence to a depth of at least 10 μm at a ϕ content of 0.07% for the 12 h liquid FLiBe exposure (approximating the C–F concentration profile as a square wave as a lower bound for C–F depth progression).

From the bulk GDMS analysis of the L12 sample (compiled in Supporting Information), we note an increase in Al, Fe, Cr, and Ni post salt exposure. It is possible that the reaction of C with minor constituents in the salt could reduce metals other than Be (e.g., eqs 7–11).
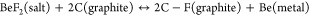
7
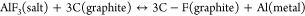
8
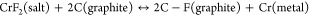
9
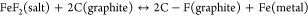
10
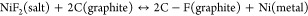
11

However, from the elemental analysis of the salt by ICP–MS and with a graphite surface area to salt of 100 m^–1^, only 31% of the content of C–F bonds reported here could be explained by reduction of these impurities from the salt (Supporting Information provides the details of this calculation). Thus, BeF_2_ reduction remains necessary for the production of C–F. Evidence of beryllium carbide is not observed in the XPS C1 spectrum, thus deposition of Be metal is postulated as a byproduct of C–F production, as shown in Figure 11.

Using the law of mass action for the chemical reaction in eq 8, the C–F surface coverage from the XPS analysis can be used in conjunction with the GDMS Be data to estimate the Gibbs’ free energy of formation (Δ*G*_f_) of C–F from C(graphite) at 700 °C. Using the coverage for sample L12, we estimate Δ*G*_f_ = −785 kJ/mol F_2_ for the semi-ionic C–F and Δ*G*_f_ = −768 kJ/mol F_2_ for the covalent C–F. Having observed that fluorination increases when the duration of exposure is increased from 12 to 240 h, these values should be considered upper bounds, as the reaction is not yet at equilibrium after 12 h. Using the coverage for sample L240, and assuming Be(metal) plating on graphite according to eq 8, Δ*G*_f_ is estimated to be −820 kJ/mol F_2_ and −828 kJ/mol F_2_ for semi-ionic and covalent, respectively. These Gibbs free energies would put C–F formation above BeF_2_ but below structural material fluorides on an Ellingham diagram. This would correspond to a C–F bond energy of >3 eV/CF, which is higher than the upper bound of 2.8 eV/CH for hydrogen chemisorption at reactive carbon sites,^1^ in line with the observation by ref (38) by XANES that C–F bonds replaced C–H bonds in graphite upon FLiNaK salt exposure. As another point of comparison, Δ*G*_f_ for CF_4_ is −393 kJ/mol F_2_ at 700 °C, suggesting that graphite fluorination is more favorable than CF_4_ evolution (thermodynamic data from ref (94)).

In order to predict CF content in graphite at thermodynamic equilibrium, the initial content of reactive carbon sites in graphite would also need to be known. As discussed in ref (1), carbon reactive sites C* exist on graphite and are responsible for hydrogen chemisorption. These sites have a finite inventory in a given graphite sample, which will depend on its prior irradiation and thermo-mechanical treatment.^1^ They are located at crystallite edges, have a Δ*G*_f_ > 0, and are more reactive than C(graphite). The spreadsheet in Supporting Information includes a sensitivity analysis of the C–F Gibbs free energy of formation on the abundance and energy of reactive carbon sites.

## 4 Discussion

### 4.1 Methods Sensitive to Graphite Surface Fluorination by Molten Salts

The nature of the chemical interaction between FLiBe and nuclear graphite is reflected in multiple features of XPS and Raman spectra, with some of them being more effective than others in characterizing the interaction.

#### 4.1.1 Interpretation of XPS Parameters Sensitive to Fluorination

The C 1s XPS can indicate the presence of fluorine–carbon bonds by subpeaks in the 285 to 289 eV range, between the main sp^2^ and sp^3^ peaks, and the π–π* peak. However, these multiple low-intensity subpeaks can be attributed to either C–F or C–O bonds or other types of defects.^25,63^ As a result, peak-fitting of the C 1s for species at sub 1% concentration may not be univocal or reproducible by other researchers performing similar analyses on the same data. For example, ref (25) assigns a subpeak at 285 eV to C–CO groups, while ref (65) assigns a subpeak at 285 to C–O ether bonds. As another example, a subpeak at approximately 288 eV can be attributed to semi-ionic C–F bonds^24−26,34,63^ or to C=O double bonds.^25,65,68,70^Table 10 illustrates two possible ways of peak fitting and interpreting the same C 1s spectrum. The two peak fittings lead to 1 and 7 for the ratio of semi-ionic C–F to covalent C–F and 10 and 9 for the sp^2^/sp^3^ ratio. The fit-to-fit variability of the C 1s subpeaks is on the order of magnitude of the point-to-point variability (Table 11), and analysis of the C 1s peak alone does not lead to a unique interpretation (Table 10). For the characterization of the sp^2^/sp^3^ ratio independently of the C 1s peak, we rely on the *D* parameter from the C KLL peak. For the characterization of C–O and C–F bonds in graphite, we rely on the O 1s and F 1s peak-fitting. While the C 1s spectrum alone does not have a unique solution for peak assignment, subpeaks C_2_–C_5_ need to be consistent with the O 1s and F 1s peak assignments; an example self-consistency check between C 1s, O 1s, and F 1s spectra is given in Supporting Information.

**Table 10:**
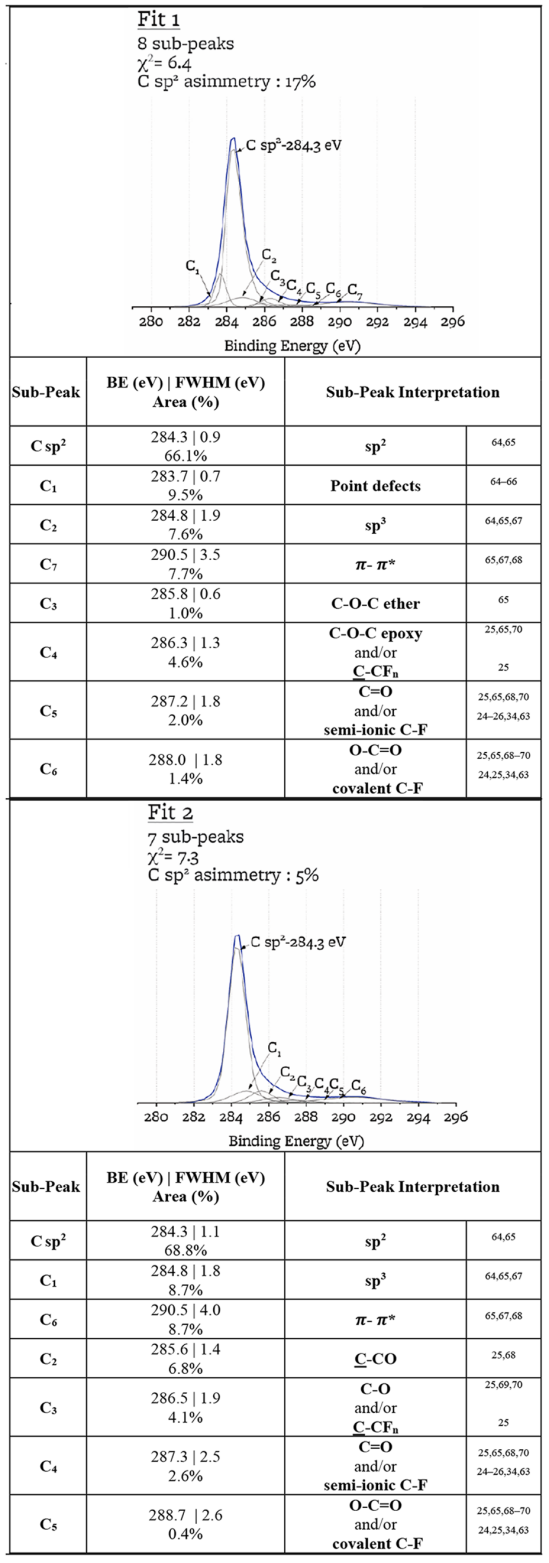
Examples of Peak-Fitting Results for C 1s Peak for Sample L240_P

**Table 11:** Variability in C 1s Sub-Peak Analysis^a^

C 1s sub-peak assignment	point-to-point variability (%)	fit-to-fit variability (%)
C sp^2^	8.5	2.0
C sp^3^	0.8	0.8
π–π*	2.1	0.7
C=O and/or semi-ionic C–F	0.6	0.4
**O–C=Oand/orcovalentC–F**	**0.4**	**0.7**
sum of OC and FC groups	3.8	1.9

a Rows where point-to-point variability is less that fit-to-fit variability are highlighted in bold.

#### 4.1.2 Interpretation of Raman Parameters Sensitive to Fluorination

Raman spectra have been widely used to extract information on the microstructural parameters of graphite at depths up to a few tens of nm.^7,46,58^ Studies using Raman spectroscopy to characterize fluorinated graphite samples have indicated multiple changes (tabulated in Table 12) consistent with a loss of crystal order in the graphite. These changes have been observed both for samples containing semi-ionic C–F^15,27,29^ and samples containing covalent C–F,^15,29,30^ produced in gas-phase reactions^27,29,30^ and liquid-phase reactions in molten salts.^15,95^ We note that similar Raman features also appear in graphite not exposed to fluorine but used in experiments involving high-temperature tribological experiments,^7,92^ ball milling,^96,97^ and irradiation^98,99^ (Table 13). Thus, Raman spectra indicate a higher content of surface defects after salt exposure that characteristically appear as a consequence of graphite-fluorination processes and need to be accompanied by other characterization techniques that verify surface fluorination.

**Table 12:** Summary of XPS and Raman Observations in Graphite Fluorination Studies

material	fluorinating agent	temperature	exposure duration	degree of fluorination ϕ	XPS observations	Raman observations	XRD observations	refs
natural graphite powder	F_2_	80–520 °C	2–10 min	up to 18%	*T* < 150 °C: F 1s shows peak for semi-ionic C–F *T* > 150 °C: F 1s shows peak for covalent C–F	increase in *I*(D)/*I*(G), FWHM(D), FWHM(G), FWHM(2G). Decrease in *I*(G′)	*T* < 150 °C: decrease in crystallite size along *c* axis *T* > 150 °C: no change in crystallite size along *c* axis	(29)
graphene films	XeF_2_	room temperature	up to 300 s	up to 25%	F 1s main peaks attributed to semi-ionic C–F	increase in *I*(D)/(*I*(G), FWHM(D), and FWHM(G)	N/A	(27)
graphite	F_2_, K_2_NiF_6_, and KAgF_4_	room temperature, 380 °C, 515 °C	1 min to 2 weeks	5% to 94%	N/A	increase in *I*(D)/(*I*(G), FWHM(D), and FWHM(G). Appearance of shoulder between D and G bands	room temperature and *T* = 380 °C: increase in interplanar distance. *T* = 515 °C: loss of crystal structure	(30)
nuclear graphite IG-110	liquid FLiBe	700 °C	12 h	<0.1%	F 1s shows peaks for semi-ionic C–F and covalent C–F. % semi-ionic C–F > % covalent C–F	increase in *I*(D)/*I*(G). FWHM(D), FWHM(G). decrease in *I*(G′) and *A*(G′). Appearance of shoulder between D and G bands	increase in interplanar distance	(15)
nuclear graphite IG-110	liquid FLiBe	700 °C	240 h	1.2(5)%	F 1s shows peaks for semi-ionic C–F and covalent C–F. % semi-ionic C–F < % covalent C–F	increase in *I*(D)/*I*(G). FWHM(D), FWHM(G). Decrease in *I*(G′) and *A*(G′). Appearance of shoulder between D and G bands	N/A	this study
nuclear graphite IG-110	cover gas above FLiBe	700 °C	240 h	0.20(12)%	F 1s shows peaks for semi-ionic C–F and covalent C–F. % semi-ionic C–F > % covalent C–F	same changes as for liquid-FLiBe-exposed but to a smaller degree	N/A	this study

**Table 13:** Examples of Graphite Surface Modification Studies That Do Not Involve Fluorination and Exhibit Defects Probed by Raman Spectroscopy That Are Similar to Those Observed in Fluorination

experiment type	temperature	atmosphere and duration	sample type	Raman observation	refs
graphite-on-graphite wear testing	600 °C	Ar gas, 1 h	nuclear graphite ET-10	increase in *I*(D)/*I*(G), FWHM(D), FWHM(G). Decrease in *I*(G′) and *A*(G′). Appearance of shoulder between D and G bands	(7,92)
stainless steel and agate ball milling	room temperature	Ar gas, up to 5000 h	graphite powder	increase in *I*(D)/*I*(G), FWHM(D), FWHM(G). Appearance of shoulder between D and G bands. Loss of D and G band distinction after 1000’s h milling	(97)
stainless steel ball milling	room temperature	vacuum, 1000 h	graphite powder	increase in *I*(D)/*I*(G), FWHM(D), FWHM(G). Appearance of shoulder between D and G bands	(96)
electron beam irradiation	room temperature	Ar gas, 5 s	isotropic graphite T-6P	decrease in *I*(D)/*I*(G). Increase in FWHM(G)	(99)
^37^Cl^+^ ion beam irradiation	room temperature to –600 °C	N/A	nuclear graphite	Increase in *I*(D)/*I*(G), FWHM(D), FWHM(G). Decrease in *I*(G′) and *A*(G′). Appearance of shoulder between D and G bands and disappearance of D and G bands at increasingly high fluences. Changes more pronounced at low temperatures and high fluences	(98)

### 4.2 Graphite–Salt Interactions

#### 4.2.1 Time-Dependence of Surface Changes

**Figure 12:**
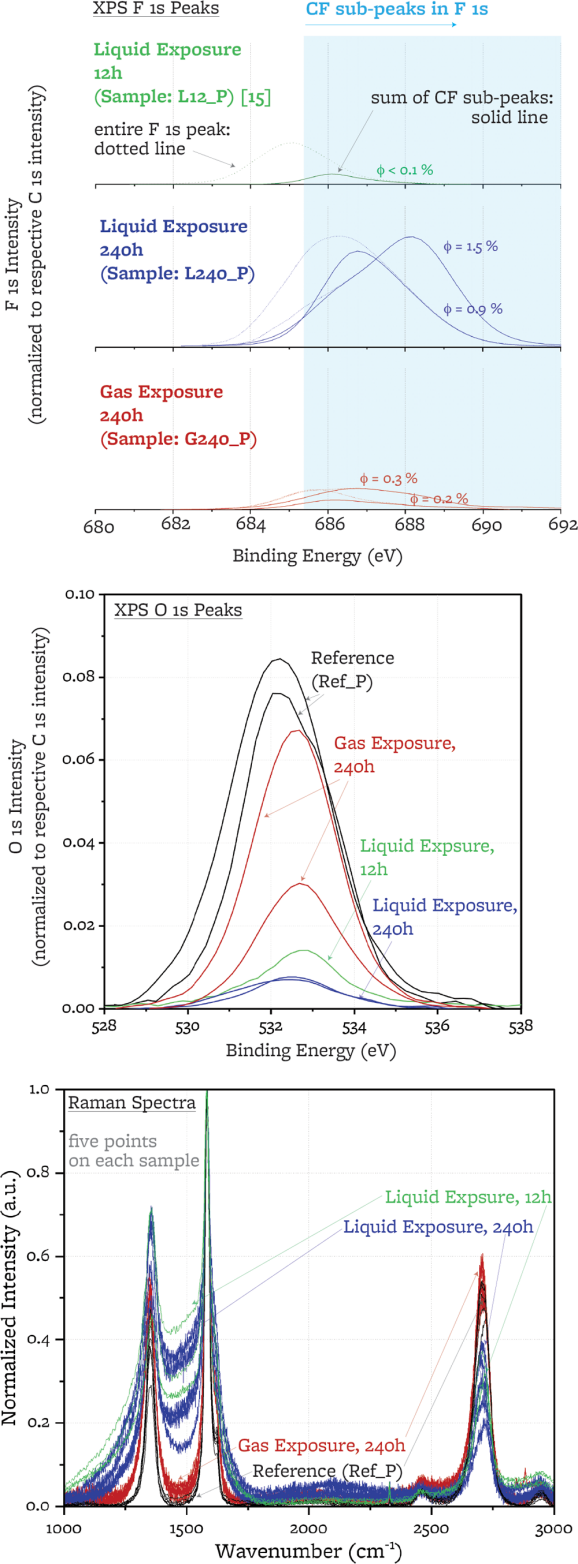
Comparison of the F 1s XPS peaks, O 1s XPS peaks, and Raman spectra of the sample exposed for 12 h^15^ and the samples exposed for 240 h (presented in this study).

The fluorine content on the graphite exposed to liquid salt for 12 h is lower than that on the graphite exposed for 240 h by a factor of 7. With longer exposure, the F 1s spectrum shifts toward higher binding energies, indicating a higher relative amount of covalent C–F as opposed to semi-ionic C–F (Figure 12 and Table 8). Surface microstructural changes, as characterized by Raman, are similar between the 12 and 240 h exposures. The oxygen content is highest in the reference sample, lower after 12 h exposure, and lowest after 240 h. The sum of oxygen and fluorine content after either duration of exposure is lower than the oxygen content of the nonexposed reference sample.

We infer, from the GDMS depth profiling, a Be metal concentration that is initially above the corresponding C–F concentration that would be produced by the reaction in eq 8.

Thus, we postulate that this reaction occurs at the salt-graphite interface and is followed by transport of Be metal and C–F into the graphite and that Be metal progression into the graphite is slower than C–F progression. Integrating the total content of Be metal, we deduce a C–F presence to a depth of at least 10 μm at a ϕ content of 0.07% for the 12 h liquid FLiBe exposure.

These observations indicate that:

(i) fluorination occurs over a time scale longer than tens of hours,
(ii) oxygen content decrease has a different time-constant than fluorination
(iii) after salt exposure, the total C–X content decreases, where C–X = C–F + C–O.
(iv) formation of semi-ionic C–F bonds occurs with faster kinetics than formation of covalent C–F bonds
(v) surface microstructural changes observable by Raman occur within 12 h of liquid exposure and do not become more pronounced with longer exposure.
(vi) C–F bonds are present to a depth of at least 10 μm.

#### 4.2.2 Comparison between Liquid-Phase and Gas-Phase Exposure

Fluorination occurs to a larger extent in the sample exposed to liquid FLiBe than to the cover gas: ϕ_liq_ = 1.2(5)%, ϕ_gas_ = 0.2(1)% (Table 8). Formation of covalent C–F and semi-ionic C–F is observed in both types of exposure. SEM/EDS shows localized fluorine-rich regions (at the microns to submicron length scale), and XPS F 1s spectra (400 μm sampling diameter) show C–F bond formation in both cover-gas-exposed and liquid-FLiBe-exposed graphite samples.

Covalent C–F bonds are hypothesized to form at crystallite edges (similarly to how oxygen has been shown to bind at crystallite edges^100−102^), in alignment with no measurable changes in the sp^2^/sp^3^ ratio (based on XPS C 1s and C KLL); however, the standard deviations on sp^2^/sp^3^ are greater than 10%, so if any changes were to occur, they would not be distinguishable. The occupancy of covalent C–F bonds on carbon edge sites is observed to be up to 10% (correcting for the fact that *C*_edge_/*C*_total_ increases after salt exposure; see Table 8). Raman spectroscopy suggests a decreased basal crystallite size *L*_a_, and thus a corresponding increase in carbon edge sites available for hosting covalent C–F bonds without increasing the sp^3^ content. C–O bond removal is also observed by O 1s XPS, with liquid-exposed having more removal of C–O bonds than the gas-exposed sample (Figure 12 and Table 8). Since C/O and C/F stoichiometry is not a priori known, we cannot precisely say if sites of oxygen removal from C–O bonds correspond in magnitude to the density of sites of C–F formation (Table 8); as an order of magnitude, we can, however, compare the remaining C–O + C–F to the initial C–O on the reference sample: for the cover-gas sample, this value is within two standard deviations of the reference sample, for the liquid-exposed samples, this value is a factor of 10 lower (more than two standard deviations away from the reference sample), and interestingly it is the same value for the 12 h and the 240 h exposures, except that the short exposure has predominantly C–O and the long exposure has predominantly C–F.

Semi-ionic C–F is hypothesized to occur as intercalates between the graphene planes. The abundance of semi-ionic F/*C*_bulk_ is observed to be up to 0.5% (correcting for the fact that *C*_bulk_/*C*_total_ decreases after salt exposure; see Table 8). Fluorine intercalates would be expected to increase the graphene layer spacing, as measured by Raman and XRD; however, at 0.5% intercalate content, these changes would be on the order of 0.007 Å (assuming 4.7 Å graphene layer spacing for semi-ionic F intercalates, as reported by ref (103)); this change would be three times smaller than one standard deviation of the interlayer spacing determined from Raman and the same order of magnitude as one standard deviation of the value determined by XRD, thus not observable by the techniques employed here. Nevertheless, surface microstructural changes, as probed by Raman, are indicative of a partial loss of crystallinity. These changes are more pronounced in the liquid FLiBe-exposed sample than the cover-gas-exposed sample (Table 7) and have been observed upon intercalation of fluorine species between graphene planes.^27,29,30^ Future studies are needed to verify the presence of semi-ionic C–F through independent techniques (e.g., by EPR or solid-state ^19^F NMR^104,105^) and to understand the degree to which such a low concentration of C–F would have an impact on irradiation behavior or macroscopic surface properties of the graphite.

From the observations above, we postulate that C–F bonds form by different mechanisms in the liquid phase than in the cover gas of the molten salt. Depth profiling (by GDMS and XPS) of the liquid FLiBe-exposed samples indicates the presence of C–F beyond the sample surface (Figures 10 and 11), a slight increase in % SI with depth, no detectable salt species beyond the first XPS sputtering step, and a shallower depth progression of the hypothesized Be metal than the depth progression of the C–F content. Since % SI and ϕ remain very different between the cover-gas-exposed sample and the depth-profiling results on the liquid-exposed sample, we postulate that the dominant mechanism of surface fluorination in the liquid occurs at the liquid–solid interface, followed by transport of the reaction products into the depth of the graphite. The transport of fluorination products into the graphite depth can occur by diffusion along the surface of crystallites, diffusion through the graphite bulk, or diffusion via gas-phase intermediaries. Future studies are needed to better elucidate the reaction mechanisms and transport mechanisms at the graphite surface and to define the corresponding time scales and spatial scales of relevance for these mechanisms. Further studies are needed to understand the length scales of the fluorination heterogeneity (as seen from the variability in degree of fluorination across XPS points and in the surface heterogeneity of SEM/EDS images) and to understand if it is linked to the initial heterogeneity of the graphite surface or if it is a manifestation of the stochastic nature of the surface fluorination process.

### 4.3 Engineering Relevance on Graphite Performance in the Reactor

The study of the effect of the long-term exposure of nuclear graphite to FLiBe at high temperatures is motivated by the usage of graphite components in MSRs and FHRs. In Table 14, we postulate how the chemical and microstructural changes at the surface of the salt and cover-gas-exposed graphite may be of engineering relevance to graphite used in nuclear reactors that employ molten salt. As shown in Table 14, we expect that surface fluorination may cause both favorable effects (decrease of wear and friction, increase in tritium chemisorption, decrease of infiltration) and unfavorable effects (increase in oxidation). The still unclear knowledge of fluorination kinetics in a reactor and the absence of tribology, hydrogen chemisorption, infiltration, or oxidation studies involving fluorinated graphite preclude us from drawing quantitative conclusions on the extent of these postulated changes in the engineering properties of graphite. To this purpose, it is recommended to replicate a subset of previous experiments on tribology,^7,8^ tritium chemisorption,^106,107^ oxidation,^59^ and infiltration^6^ using graphite that has been surface-fluorinated by salt exposure.

**Table 14:** Postulated Impact of Surface Fluorination of Graphite by Liquid FLiBe Salt and Its Cover Gas

phenomenon	relevance to FHR/MSR operation and waste management	fluorination changes with impact on the phenomenon	
wear and friction in salt-lubricated environment	• FHR graphitic fuel elements slide and roll against each other while immersed in salt• wear and friction resulting from the contact can cause deterioration of fuel elements, dust generation, and increase of core residence time^7,8^ • wear and friction lowered when a lubricious carbon film forms on graphite surface^7^	• decrease in *L*_a_ and oxygen release (decreasein Ω) upon fluorination cause increase in the number of edge sites available for C–C bonding• formation of covalent C–F leads to passivation of edge sites	


tritium chemisorption	• MSR/FHR graphite can chemisorb tritium during reactor operation• this leads to a decrease of the tritium source term in the core but also increases tritium activity in the spent fuel and may require tritium desorption as the components are extracted from the core^3,4^	• decrease in *L*_a_ and oxygen release (decrease in Ω) cause increase in the number of edge sites available for C–^3^H bonding• formation of covalent C–F leads to passivation of edge sites, making them unavailable to tritium	

graphite oxidation	• MSR/FHR graphite can be oxidized by oxide impurities in the salt (chronic oxidation) and by air or oxygen ingress in accident events (acute oxidation)• oxidation can cause deterioration of fuel elements and impact isotope transport	• decrease in *L*_a_ increases number of edge sites• formation of semi-ionic C–F may increase interlayer spacing	

salt infiltration	• salt can infiltrate graphite porosity under kPa–MPa pressures^5,6^ • infiltration is increased if salt starts wetting graphite• infiltration will change pebble buoyancy in the core, can impact heat transfer and mechanical properties, and cause carry-over of salt into spent fuel storage^5^	• graphite fluorination reported to limit wetting (i.e., higher contact angle) by fluoride salts^72^	


## 5 Conclusions

In nuclear reactors that employ graphite components and molten fluoride salts, characterizing the chemical and microstructural changes of graphite caused by salt-graphite interactions is relevant to assessing the performance of nuclear graphite during reactor operation and to predicting graphite conditions upon its discharge from the reactor after one to tens of years of molten fluoride salt exposure of the graphite. This study advances the understanding of the chemical interactions between molten FLiBe salt and nuclear graphite in MSR and FHR advanced nuclear reactors.

Chemical and microstructural changes occur in nuclear graphite upon exposure to FLiBe salt for 240 h at 700 °C, with samples exposed to the liquid FLiBe and samples exposed to the cover gas above molten salt, based on characterization of the samples performed by SEM/EDS, surface XPS, depth profiling XPS, and Raman spectroscopy, and reanalysis of prior GDMS depth profiling and XPS data of a 12 h exposed sample.

We identify both semi-ionic and covalent C–F bond formation on the surface, to differing degrees, in the salt-exposed and cover-gas-exposed samples. We conclude that C–F bonds form by different mechanisms in the liquid phase than in the cover gas of the molten salt. Depth-profiling XPS confirms the formation of C–F bonds beyond the first few tens of nanometers from the surface. Reinterpretation of GDMS depth profiling of the 12 h exposed sample, in conjunction with XPS analysis, indicates a C–F presence to a depth of at least 10 μm at a ϕ content of 0.07% for the 12 h liquid FLiBe exposure. Superficial profiling (<0.1 μm by XPS and <5 μm by GDMS) indicates increasing F content with depth while showing no progression of salt species beyond the surface of the graphite. Future studies are warranted for the transport of C–F species in graphite at high temperatures. C–F formation is accompanied by surface microstructural changes and removal of C–O groups.

Further studies are needed to establish the relationships among chemical and surface microstructural modifications in graphite upon exposure to molten salt and the cover gas above it, to confirm the formation of covalent and semi-ionic C–F, and to develop a mechanistic description for the formation of covalent and semi-ionic C–F with exposure to salt and to the cover-gas above the salt. Given the difference between the 12 and 240 h exposures, future studies are warranted on the reaction kinetics of fluorination.

Graphite components in MSRs and FHRs are exposed to fluoride salts for durations up to tens of years, i.e., 2–3 orders of magnitude longer duration than in this experiment. The increase in C–F bond concentration observed when increasing the exposure duration from 12 to 240 h suggests that further fluorination might occur with longer exposures. The observation of a decrease in the total C–F + C–O content with time seems to suggest that fluorination might not continue indefinitely but may be limited by the pre-existing oxygen content or reactive carbon sites. Future studies of surface modifications by salt exposure of neutron-irradiated graphite, which would contain a higher density of defects and reactive carbon sites, are warranted.

The possible relevance of the fluoride-salt-induced surface modifications to graphite engineering for nuclear reactors may include effects such as modification of friction coefficient and wear rate, modification of tritium chemisorption in graphite from molten salt during nuclear reactor operation and of the effectiveness of thermal desorption for decontamination of tritium from graphite post discharge from the rector, effects on chronic and acute graphite oxidation by dry air, steam, or other oxidants, and changes in salt wetting and consequently changes in salt infiltration in the pores of graphite. Future studies are warranted to verify if any of these postulated effects are of engineering significance at the relatively low surface concentration of 1% C–F at the graphite surface.

## Supporting Information Available

Additional EDS point spectra of the polished surface of the sample exposed to liquid FLiBe and a link to Mendeley repository containing additional SEM/EDS images of all samples, peak-fitted Raman and XPS spectra, and raw Raman, XPS, and GDMS data (PDF)
