# Supplementary material for: Surface Fluorination of Nuclear Graphite Exposed to Molten 2LiF–BeF2 (FLiBe) Salt and Its Cover Gas at 700 °C
Source: ACS Appl Eng Mater. 2024 Jun 17;2(6):1483–502. doi: 10.1021/acsaenm.3c00764 (PMC11217946; doi:10.1021/acsaenm.3c00764)
Supplement: Supplementary file 1 — em3c00764_si_001.pdf [file em3c00764_si_001.pdf]

Supporting Information for:
Surface fluorination of nuclear graphite exposed to molten $2LiF\text{-}BeF_2$ (FLiBe) salt and its cover gas at 700 °C

L. Vergari[a,b], H. Wu [c,d], R.O. Scarlat[a,*]

[a] Department of Nuclear Engineering, University of California Berkeley, 2521 Hearst. Ave, Berkeley 94720, USA [b] Department of Nuclear, Plasma and Radiological Engineering, University of Illinois Urbana-Champaign, 104 S. Wright Street, Urbana 61801, USA

[c] Department of Engineering Physics, University of Wisconsin – Madison, 1500 Engineering Drive, Madison 53706, USA

[d] Canadian Nuclear Laboratories, 286 Plant Rd, Chalk River, ON, K0J 1J0, Canada

[*] Corresponding author, scarlat@berkeley.edu

1. EDS Point Spectra

Figure S1 displays EDS point spectra collected on selected features on the polished surface of the liquid-FLiBe exposed sample (L240_P).

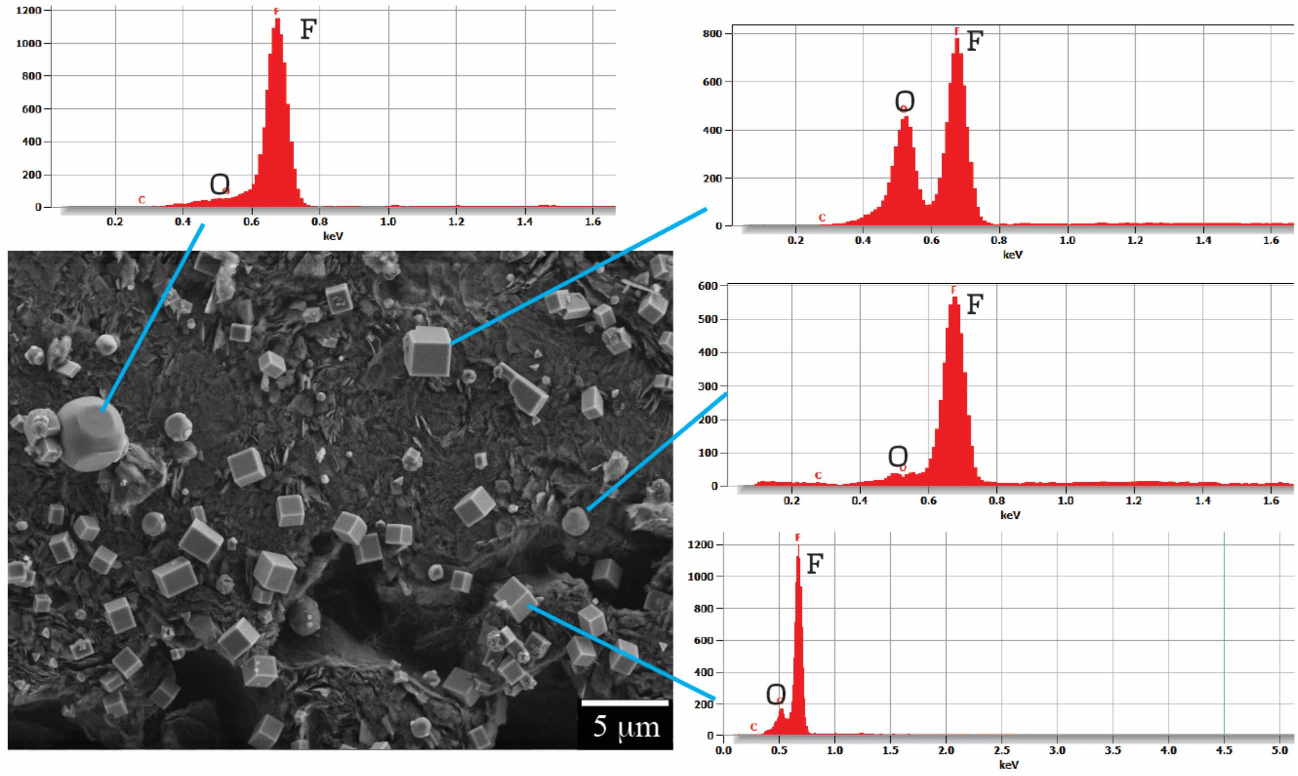

Figure S1: EDS point spectra of the polished surface of the sample exposed to liquid FLiBe (L240_P). Collected using a Zeiss LEO 1530 at an accelerating voltage of 10 kV

2. Supplementary SEM Micrographs

Included in a Mendeley repository available at: https://doi.org/10.17632/22tgxbphjd.1.

3. Supplementary EDS Maps

Included in a Mendeley repository available at: https://doi.org/10.17632/22tgxbphjd.1.

4. XPS

A spreadsheet including sample superficial composition from XPS survey spectra and peak-fitted XPS spectra is included in a Mendeley repository available at: https://doi.org/10.17632/22tgxbphjd.1.

5. Raman Spectroscopy

A spreadsheet including peak-fitted Raman spectra is included in a Mendeley repository available at: https://doi.org/10.17632/22tgxbphjd.1.

6. GDMS and Thermodynamic Calculations

Spreadsheet including GDMS characterization for sample L12 and thermodynamic calculations of the Gibbs free energy of C-F formation are included in a Mendeley repository available at: https://doi.org/10.17632/22tgxbphjd.1.
